# Evolution of the Brassicaceae‐specific *MS5‐Like* family and neofunctionalization of the novel *MALE STERILITY 5* gene essential for male fertility in *Brassica napus*

**DOI:** 10.1111/nph.17053

**Published:** 2020-11-23

**Authors:** Xinhua Zeng, Hao Li, Keqi Li, Rong Yuan, Shengbo Zhao, Jun Li, Junling Luo, Xiaofei Li, Hong Ma, Gang Wu, Xiaohong Yan

**Affiliations:** ^1^ Oil Crops Research Institute of the Chinese Academy of Agricultural Sciences/Key Laboratory of Biology and Genetic Improvement of Oil Crops Ministry of Agriculture Wuhan 430062 China; ^2^ Department of Biology the Huck Institutes of the Life Sciences the Pennsylvania State University University Park PA 16802 USA

**Keywords:** *Brassica MS5*, lineage‐specific gene, male fertility, neofunctionalization, new gene, SUN protein, telomeric dynamics

## Abstract

- New genes (or lineage‐specific genes) can facilitate functional innovations. *MALE STERILITY 5* (*MS5*) in *Brassica napus* is a fertility‐related new gene, which has two wild‐type alleles (*BnMS5^a^* and *BnMS5^c^*) and two mutant alleles (*BnMS5^b^* and *BnMS5^d^*) that could induce male sterility.
- Here, we studied the history and functional evolution of *MS5* homologs in plants by phylogenetic analysis and molecular genetic experiments.
- We identified 727 *MS5* homologs and found that they define a Brassicaceae‐specific gene family that has expanded partly via multiple tandem gene duplications and also probably transpositions. The *MS5* in *B. napus* is inherited from a basic diploid ancestor of *B. rapa*. Molecular genetic experiments indicate that *BnMS5^a^* and *BnMS5^c^* are functionally distinct in *B. napus* and that *BnMS5^d^* can inhibit *BnMS5^a^* in *B. napus* in a dosage‐dependent manner. The BnMS5^a^ protein can move in coordination with meiotic telomeres and interact with the nuclear envelope protein SUN1, with a possible crucial role in meiotic chromosome behavior.
- In summary, *BnMS5* belongs to a Brassicaceae‐specific new gene family, and has gained a novel function that is essential for male fertility in *B. napus* through neofunctionalization that has likely occurred since the origin of *B. rapa*.

## Introduction

Genes experience dynamic evolutionary processes of origination, diversification and loss. New genes (or lineage‐specific genes) refer to those that were ‘born’ at a particular time in a species or lineage and had not existed previously (Chen *et al*., 2013). New genes can acquire novel functions and are important drivers of adaptive evolution that contribute to the establishment of novel molecular processes (Cardoso‐Moreira & Long, 2012; Weng *et al*., 2012), with profound impact on evolution of physiology and development (Zhang *et al*., 2002; Parker *et al*., 2009; Chen *et al*., 2010; Carelli *et al*., 2016). New genes could arise through various mechanisms, such as duplications, *de novo* originations, domain shuffling and incorporation of mobile elements (Kaessmann *et al*., 2009; Long *et al*., 2013). Specifically, gene duplication is prevalent, and occurs at dramatically different scales, such as whole genome duplication (WGD) and small‐scale duplications (Semon & Wolfe, 2007; Hanada *et al*., 2008; Vanneste *et al*., 2014; Ren *et al*., 2018). Notably, transposition can be an important mechanism for small‐scale duplication (Freeling, 2009). Moreover, gene duplicates are a primary source of genetic novelty (Ohno, 1970; Kaessmann, 2010; Chen *et al*., 2013) and can undergo three possible functional diversifications: pseudogenization (nonfunctionalization), subfunctionalization and neofunctionalization (Lynch & Conery, 2000; Zhang, 2003). Functional diversification of duplicate genes often plays an important role in the generation of lineage‐specific traits (Wapinski *et al*., 2007; Han *et al*., 2009).

Unlike gene duplication, *de novo* formation of genes with novel sequences occurs relatively infrequently (Kaessmann, 2010; Li *et al*., 2016; McLysaght & Hurst, 2016); nevertheless, such *de novo* genes contribute to functional innovation and also might involve interactions with pre‐existing genes that function during the same developmental stages and/or under the same environmental conditions. In addition, the emergence of *de novo* genes might bring evolutionary innovations to a species for its adaptation to new environments (Long *et al*., 2003; Kaessmann *et al*., 2009). However, there have not been many studies of functions of *de novo* genes, especially in plants.

*Brassica napus* (genome AACC, 2*n* = 38) is an allotetraploid member of the Brassicaceae (formerly Crucifers), which has *c.* 3700 species, with several important vegetables (i.e. cabbage, broccoli, cauliflower and radish) and the model plant *Arabidopsis thaliana*, and has been proposed as a model family for evolutionary studies (Al‐Shehbaz *et al*., 2006; Huang *et al*., 2016). In *Brassica*, three diploids (referred to as the basic diploids) have been characterized: *B. rapa* (AA genome, *n* = 10), *B. nigra* (BB, *n* = 8) and *B. oleracea* (CC, *n* = 9); multiple natural hybridizations among members of these basic diploids (Parkin *et al*., 1995; Chalhoub *et al*., 2014) resulted in three allotetraploids, *B. juncea* (AABB, *n* = 18), *B. napus* (AACC, *n* = 19) and *B. carinata* (BBCC, *n* = 17) (Nagaharu, 1935), leading to further genomic evolution during allopolyploid speciation (Marhold & Lihová, 2006). Moreover, *Brassica* and related genera belong to a tribe called Brassiceae, within Brassicaceae (Lysak *et al*., 2005). Genomic collinearity analysis demonstrated that each of 24 conserved chromosomal blocks of *A. thaliana* often corresponds to three syntenic copies in each basic *Brassica* diploid, supporting an ancient whole‐genome triplication (WGT) shared by *Brassica* and close relatives in Brassiceae (Schranz *et al*., 2006; Wang *et al*., 2011; Cheng *et al*., 2013). Furthermore, *Brassica* and others in Brassiceae share even older WGDs (called α and β) with other members of Brassicaceae including *A. thaliana* (Bowers *et al*., 2003; Schranz *et al*., 2006; Wang *et al*., 2011; Liu *et al*., 2014).

In *B. napus,* the *MALE STERILITY 5* (*MS5*) (*BnMS5*) locus defines a novel genetic system (named TE5ABC) controlling male fertility with three genetically different lines (TE5A, TE5B and TE5C), each with different combinations of *BnMS5* alleles (Xin *et al*., 2016; Zeng *et al*., 2016) (Supporting Information Fig. S1). Specifically, the *BnMS5^a^* allele is dominant over *BnMS5^d^*, but *BnMS5^c^* is recessive to *BnMS5^d^*; thus, *B. napus BnMS5^a^MS5^a^* lines are referred to as restorer lines because they restore fertility when crossed with *BnMS5^d^MS5^d^* lines. However, *BnMS5^c^MS5^c^* lines are called temporary maintainer lines because *BnMS5^c^MS5^d^* plants are male sterile (Lu *et al*., 2013; Xin *et al*., 2016; Zeng *et al*., 2016). Another allele, *BnMS5^b^*, was derived from *BnMS5^a^* by mutagenesis and confers male sterility when homozygous (Xin *et al*., 2016).

In brief, both the *BnMS5^a^* and *BnMS5^c^* alleles are likely functional, whereas *BnMS5^d^* is defective in male fertility (Fig. S1a). The fact that *BnMS5^c^MS5^d^* plants are sterile indicates that *BnMS5^c^* is unable to overcome the defect of *BnMS5^d^*. Intriguingly, our preliminary study indicated that *BnMS5* and its homologs are detected only in members of Brassicaceae, but not other plants or nonplant organisms, suggesting that they are Brassicaceae‐specific new genes. The *BnMS5‐*dependent genetic male sterility (GMS) system provides an excellent opportunity to study the evolution of new genes that play crucial roles in fitness. In this study, we analyzed the evolutionary history of *MS5* homologs and showed how a recently originated *MS5* gene rapidly evolved a novel function likely within the *Brassica* genus, became integrated into an existing cellular network and impacted male development in *B. napus*.

## Materials and Methods

### Plant materials

*Arabidopsis thaliana* (Col‐0) was used for β‐glucuronidase (GUS) analysis and genetic transformation. Fifty *Brassica napus* inbred lines and 30 *B. rapa* varieties were used for genotype frequency analysis (Tables S1, S2). Twenty‐two *Brassica* accessions were from the Centre for Genetic Resources, Plant Genetic Resources (CGN‐PGR; the Netherlands), with extensive genetic diversity (Table S3). Transgenic *A. thaliana*, *B. rapa* and *B. napus* plants were planted in the glasshouse. Nontransgenic plants were planted under normal farming conditions in Hubei, China.

### Identification of *MS5* homologs in plants

Eighty‐three angiosperms and other green plants were used for *MALE STERILITY 5* (*MS5*) homologs search (Table S4), with their genomes downloaded from phytozome v.12 (Goodstein *et al*., 2012), ConGenIE (Nystedt *et al*., 2013), the *Brassica* database (Cheng *et al*., 2011), the *B. napus* genome database (Chalhoub *et al*., 2014), the *Barbarea vulgaris* Genome Database (Byrne *et al*., 2017), the *Cardamine hirsuta* genetic and genomic resource (Gan *et al*., 2016), the maca genome (*Lepidium meyenii*) (Zhang *et al*., 2016), *Raphanus sativus* Genome DataBase (Kitashiba *et al*., 2014), RadishDB (Moghe *et al*., 2014), and NCBI (Agarwala *et al*., 2018). BnMS5^a^ (accession ID in NCBI: ANN45948) was used for PSI‐Blast (*E*‐values ≤ 1×10^−10^). According to the Pfam (Finn *et al*., 2016) and SMART (Letunic *et al*., 2015) databases, targets from PSI‐Blast search with a MS5 domain (*E*‐value ≤ 1×10^−10^) were designated as *MS5* homologs here (Table S5). We also re‐annotated 25 of the 727 *MS5* homologs based on their genomic sequence and closely‐related homologs (Dataset S1).

### Genome synteny analysis

For the synteny analysis of homologs in each lineage (Tables S6, S7), MCScanX was used to identify inter‐/intraspecies syntenic blocks by using BlastP results and chromosomal locations of genes (match_score:50, match_size: 10, gap_penalty: −1, overlap_window: 5, *E*‐value: 1 × 10^−10^, max gaps: 25) (Wang *et al*., 2012). For the analysis of the *MS5* locus‐related genomic regions in eight Brassicaceae genomes, the results were derived and visualized in a synteny analysis tool of Brassicaceae species in the *Brassica* database (http://brassicadb.org/brad/searchSynteny.php) (Cheng *et al*., 2011).

### Isolation of putative *MS5* ortholog sequences from *Brassica* species

For full‐length CDS amplification, total RNA from young buds were used in reverse transcription‐PCR. According to conserved sequences of the ortholog *MS5* from *B. napus* and *B. rapa*, primers were designed to amplify *MS5* CDS from 22 different *Brassica* species or accessions (Tables S3, S8). Different *MS5* sequences obtained from one accession using the same primer pair were named with −1 and −2.

### Sequence alignment and phylogenetic analysis of *MS5* homologs

Protein sequences of *MS5* homologs were aligned by Mafft v.7.429 (Katoh & Standley, 2013) with option '‐‐auto', manually adjusted using Mega v.7.0.26 (Kumar *et al*., 2016), and then were trimmed by trimAL v.1.4 (Capella‐Gutierrez *et al*., 2009) with options '–gt 0.3'. The maximum‐likelihood (ML) tree was built based on the alignment of coding sequences (nucleotide), which were converted from the corresponding protein alignment by Pal2nal v.14 (Suyama *et al*., 2006). A total of 951 sites of the aligned region of 727 CDS sequences of *MS5* homologs were used for phylogenetic inferences (Dataset S2). IQ‐Tree v.1.6.12 (Nguyen *et al*., 2015) was applied to reconstruct all of the ML trees, with the evolutionary model (GTR + F + ASC + R6) specified by modelfinder (Kalyaanamoorthy *et al*., 2017) and ultrafast bootstrap approximation (UFBoot) of 1000 bootstrap replicates (Hoang *et al*., 2018).

### Genetic transformation of plants

For functional complementation, full open reading frames (ORFs) of *BnMS5^c^*, *BnMS5^a^*, *BnMS5^d^* and *BnMS5II* in *B. napus*, *BoMS5II‐1/‐2* in *B. oleracea* and *BniMS5III* in *B. nigra* were cloned into the PBI121S binary vector (Table S9). To knock down the *MS5* expression, a 400‐bp fragment of the *BnMS5^a^* cDNA sequence were inserted into the pART27 binary vector using the pHANNIBAL intermediate vector (Yan *et al*., 2012) (Table S8). For analysis of expression pattern, *c*. 1100‐bp upstream regions of the *BnMS5^a^*, *BnMS5^c^* and *BnMS5II* genes were amplified and cloned into pBI101, generating promoter‐GUS fusion constructs. All of the above‐mentioned constructs were transformed into the *Agrobacterium tumefaciens* strain GV3101, which was used for transformation of *A. thaliana* through the floral dip method (Clough & Bent, 1998); and transformation of *B. napus*, *B. rapa* and *B. oleracea* was performed as described previously (Yan *et al*., 2012).

### Double‐immunolabeling experiment

The probe used for fluorescence in situ hybridization (FISH) was from the pWY96 vector, which contained telomeric repeats (Yan *et al*., 2017). FISH and immunofluorescence were performed as described previously (Yan *et al*., 2016). Primary images were captured by a confocal microscope. The final images were merged using Adobe photoshop 5.0 software.

### Gene expression analysis

For mRNA expression analysis, total RNA was isolated from various tissues. Quantitative real‐time (qRT)‐PCR was conducted in triplicate with the CFX96 real‐time system (Bio‐Rad). The results were analyzed using cfx manager software with the 2^−ΔΔCT^ method (Livak & Schmittgen, 2001), with the *B. napus* endogenous reference gene cruciferin A (*CruA*) as the control for normalization (Wu *et al*., 2010). For Western blotting, proteins were extracted from transgenic 6449 leaves with RIPA lysis buffer (Beyotime, Haimen, China). The protein concentration was quantified using a protein assay kit (Bio‐Rad). Proteins were separated by 10% SDS‐PAGE and transferred onto a polyvinylidene fluoride membrane (Millipore). The primary antibody was anti‐BnMS5. Goat anti‐rabbit IgG (H + L) conjugated to horseradish peroxidase was used as the secondary antibody. Immunoblots were visualized using the Pierce ECL detection system.

### Droplet digital PCR (ddPCR)

In order to estimate transgene copy number, both single and duplex ddPCR were performed in a QX200 ddPCR system (Bio‐Rad) as described previously (Xu *et al*., 2016; Collier *et al*., 2017). The reaction mix is partitioned into thousands or millions of tiny individual reaction droplets for PCR runs by water‐oil emulsion. Reactions involving < 8000 droplets per 20 μl mixture were excluded from subsequent analysis. The number of positive droplets and the total number of droplets were determined using QuantaSoft (Bio‐Rad). Transgene copy number was determined by the ratio of exogenous genes to reference genes.

### Yeast two‐hybrid system and GST pull‐down analysis

In order to test for protein interactions, the Gal4‐based Matchmaker Gold Yeast Two‐Hybrid (Y2H) System (Clontech, Palo Alto, CA, USA) was used. *BnMS5^a^*, *BnMS5^d^*, *BnMS5^c^* and truncated *BnMS5^a^* mutants with different deletions were introduced into the pGBKT7 plasmid as baits, and *SUN1*, *SUN1Δ1* and *SUN1Δ2* were cloned into the pGADT7 vector as preys (Table S8). The bait and prey constructs were co‐transformed into Y2H Gold yeast cells and selected on synthetic dropout nutrient medium SD/‐Trp‐Leu‐His‐Ade plates with Aureobasidin A (AbA) and X‐α‐galactosidase (X‐α‐gal). For glutathione S‐transferase (GST) pull‐down analysis, plasmids pET28a (containing His tag), pET28a::SUN1, pGEX‐6p‐1 (containing GST tag), and pGEX‐6p‐1::BnMS5^a^, were transformed into the *Escherichia coli* strain BL21 (DE3), respectively, and the protein expression was induced with 0.3 mM IPTG at 30°C for 5 h. Equal amounts of His‐SUN1 and GST‐BnMS5^a^ sonicated cell lysates were mixed with GST resin (GenScript, Piscataway, NJ, USA) and incubated at 4°C overnight, and then the mixtures were washed and eluted with elution buffer. The collected proteins were separated by 10% SDS‐PAGE and immunoblotted with anti‐His and anti‐GST antibodies (Proteintech, Rosemont, IL, USA).

## Results

### Identification and phylogenetic analysis of *MS5* homologs

The *BnMS5^a^* sequence was used to search for its homologs in the genomes of 83 plant species with an additional analysis for protein domain (Table S4), resulting in the identification of 727 homologs (i.e. 727 different gene loci) in 23 Brassicaceae species, but not in the other plants, nor in nonplant organisms (Fig. S2; Dataset S1). The results suggest that *BnMS5* and its homologs likely define a Brassicaceae‐specific gene family; it will be referred to as the *MS5‐Like* family hereafter. A recent phylogenetic study divided Brassicaceae species into six clades (ABCDEF), and the 23 species herein belong to Clades A (e.g. *A. thaliana* and *Camelina sativa*), B (e.g. *B. napus* and *B. rapa*), D (*Arabis alpina*) and F (*Aethionema arabicum*) (Huang *et al*., 2016) (Fig. S2). The numbers of detected *MS5‐Like* family members in a species vary widely, even among relatively closely related species in the same phylogenetic clade, from 18 homologs in *A. thaliana* to 119 in *C. sativa* and four in *Leavenworthia alabamica* (Table S4), despite the fact that both the last two species experienced a lineage‐specific polyploidy event, respectively (Haudry *et al*., 2013; Kagale *et al*., 2014). Likewise, 94 *MS5* homologs are found in *B. napus*, whereas only 41 and 20 homologs are found in the basic diploids, *B. rapa* and *B. oleracea*, respectively, with only two homologs in the slightly more distant *Schrenkiella parvula*. The *MS5‐Like* family might have experienced uneven gene duplication and/or gene loss events across Brassicaceae.

**Fig. 1:**
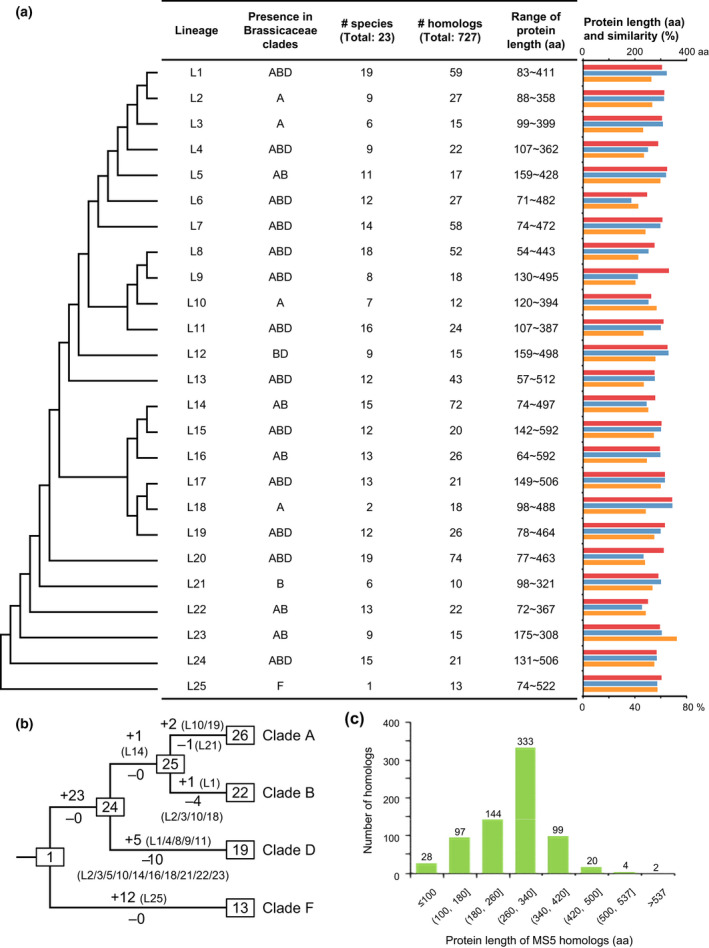
Evolutionary relationships among 25 *MALE STERILITY 5* (*MS5*)*‐Like* homolog lineages and copy number variations in each lineage. (a) On the left is a simplified *MS5‐Like* family tree showing phylogenetic relationships among the 25 homolog lineages, derived from the phylogenetic tree in Supporting Information Fig. S3. In the table, the first column shows lineage numbers, and L indicates lineage. The second column shows the Brassicaceae clade(s) that contain homologs belonging to specific lineages; the Brassicaceae clade(s) are defined according to a recently published Brassicaceae phylogeny (Huang *et al*., 2016). The third column shows the number of species that possess homologs in the specific lineage. The fourth column shows the number of homologs in each lineage. The fifth column shows the range of protein lengths in each lineage. On the right of the table is a histogram for the lineages, showing median protein length (in amino acids, aa; red bars), the length of conserved protein region (aa, blue bars) and median of percentage amino acid sequence similarity of conserved protein region (%, orange bars). For detailed data see Tables S4, S5, S10. (b) A brief tree with relationships among the clades ABCD, showing *MS5‐Like* homolog copy numbers in the last common ancestors (LCAs) of clades A, B, C and D, respectively, and their gain/loss numbers starting from one copy in the LCA of Brassicaceae, and then 13 copies in the LCA of Clade F and 24 copies in the LCA of Clade A/B/D. Numbers in parentheses indicate the lineages where gains/losses are detected. (c) Protein length distribution of the 727 *MS5* homologs identified in this study. A partial MS5 domain was detected in the 26 proteins with < 100 aa, with sufficiently high level of sequence similarity.

In order to address the evolutionary history of the *MS5‐Like* family, we constructed the phylogenetic tree of all 727 *MS5* homologs; a comparison of the gene tree with the species phylogeny supports the classification of *MS5‐Like* genes into 25 lineages (Figs 1a, S3, S4; Table S4), as described next. The *MS5‐Like* gene tree indicates that all 13 *Aethionema* genes are closely related to each other, forming a well‐supported clade; the simplest interpretation of the *MS5‐Like* gene tree topology is that all detected *Aethionema MS5‐Like* genes form a sister clade (named as Lineage 25) to other *MS5‐Like* genes (Fig. S2). Among the other genes, a well‐supported clade (bootstrap ≥ 60) is hypothesized to be derived from a single gene in the last common ancestor (LCA) of the clades A, B and D, and named as a separate lineage, if the said clade meets one of the following criteria, and it cannot be further divided into smaller clades that still meet one of these criteria: (1) containing genes from species in clades A and/or B, as well as from Clade D; or (2) being a sister clade to a clade described in (1). Thus we hypothesized that the *MS5‐Like* gene family originated as a single copy gene in the LCA of Brassicaceae, and then the gene family expanded extensively during the respective histories of *A. arabicum* and the ABD clades after the divergence of Clade F from the others. Specifically, the copy number increased to ≥ 13 in *A. arabicum* (L25); and in the LCAs of clades A, B and D, the copy number increased to 24 (one for each of lineages 1–24), as the *MS5* homologs from Clade D, *A. alpina*, do not form a monophyletic group, nor do the gene from clades A or B. The phylogeny of *MS5*‐*Like* family also indicates that lineages 1–24 each contains 10 to 74 homologs from two to 19 species (Fig. 1a; Table S10). We further estimated gene copy number changes from the LCA of Brassicaceae to that of clades A, B, D or F by a comparison of the *MS5‐Like* family tree with the Brassicaceae species tree (Fig. S2). Taken together, these results showed that the *MS5*‐*Like* family experienced ≥ 43 gains and 15 losses, respectively, from the LCA of Brassicaceae to the LCAs of clades A, B and D (Fig. 1b).

**Fig. 2:**
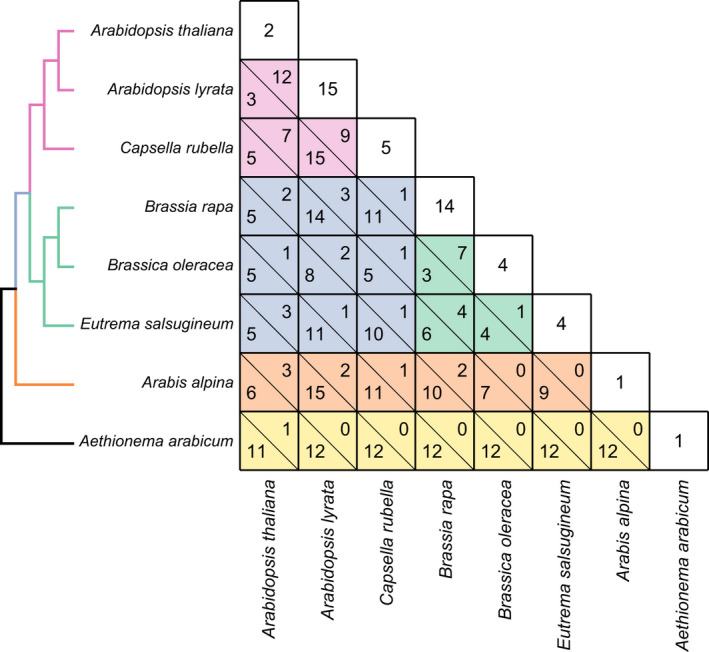
Analysis of chromosome positions of *MALE STERILITY 5* (*MS5*) homologs in eight Brassicaceae species. In the matrix here are numbers of *MS5* homologs in tandem repeats detected within species (undivided boxes along the diagonal), and number of syntenic and nonsyntenic homolog pairs (divided boxes) between two of the eight species. In each divided box, the upper right number indicates that of syntenic homolog pairs and the bottom left number indicates that of nonsyntenic homolog pairs between the species. Detailed information is summarized in Supporting Information Table S7. Red background indicates comparisons between species within Clade A; green, comparisons between species within Clade B; blue, comparisons between species from clades A and B; orange, comparisons between species from clades A and D, or from clades B and D; yellow, comparisons between species from Clade F and the other clades, and these comparisons are between homologs from different lineages. A species tree based on the phylogenetic relationship of the eight species is shown on the left (Huang *et al*., 2016).

In order to further investigate the history of *MS5* homologs, we examined the chromosomal locations of the *MS5* homologs and found that a subset of them form tandem repeats in closely spaced chromosome regions, suggesting that they originated by tandem duplication (46 tandem duplications are detected in eight representative species; Fig. 2; Tables S6, S7). We then investigated homologous chromosome regions using pairwise interspecific synteny among the eight species, and found that 64 interspecific pairs of homologs (in 20 of the 25 *MS5* lineages) are located in syntenic chromosome regions (Fig. 2; Table S7), suggesting that they could have maintained the ancestral chromosome locations. However, 132 other homolog pairs of the *MS5*‐*Like* family are not located in any interspecific syntenic region. Moreover, only one *MS5* homolog from *A. arabicum* has a detected syntenic homolog in other species, which is a homolog from *A. thaliana* (Table S7). The observation that a majority of *MS5* homologs lack syntenic relationships with other related genes suggests that their current chromosomal positions is less likely to have resulted from WGD, but instead possibly be the consequence of chromosomal segment reshuffling, transposition and/or other unknown mechanisms. Transposable elements (TEs) are mobile genetic elements that are prevalent across plant genomes and have various crucial cellular functions, and could be co‐opted for *cis*‐regulation in host regulatory pathways (Chuong *et al*., 2017; Underwood *et al*., 2017). Thus, we examined genomic sequences near each of the 18 *A. thaliana* homologs and found that the genomic sequences of 10 homologs contain or are closely adjacent (< 1 kb) to a total of 26 different transposon elements (TEs), including members from RC/Helitron, DNA/HAT, LINE/L1, DNA/MuDR and DNA/Harbinger transposon superfamilies (Table S11). These results supported transposition possibly being a mechanism for the expansion of the *MS5‐Like* family, potentially providing a new expression pattern to the *MS5* homologs.

The possible contribution of transposition‐mediated expansion of the *MS5‐Like* family suggests that *MS5* homologs might vary in coding region length dramatically. An inspection of the predicted MS5 protein lengths found a range from 54 to 592 amino acids (aa) (median value 290 aa) (Fig. 1a,c; Table S10), with the median value of protein lengths in each of the 25 lineages from 247 to 343 aa. We further examined the genomic sequences of 26 members with sequence < 100 aa, and found that in each of them a partial MS5 domain with a score higher than the threshold could be detected, and they could not be re‐annotated as longer protein sequences. In addition, the conserved protein regions of each lineage have 186 to 344 sites and contain a MS5 protein domain (formerly named as DUF626), with the median amino acid sequence similarities of the conserved regions in each lineage from 40.38% to 72.22%. We also detected the *Ka/Ks* ratio between homologs of *A. thaliana* and *A. lyrata* in each homolog lineage which contains homologs from both species, and found that the medium *Ka/Ks* value of 52 homolog pairs was 0.67 (Table S12). Additionally, homologs from these two species often are expressed in anthers and siliques at low to moderate levels; moreover, some *A. thaliana* homologs with adjacent TE show different expression patterns from the homologs without TE in the same lineage (Table S11) (Klepikova *et al*., 2016). The sequence and expression results suggest that the *MS5‐Like* family is highly dynamic, with functionally divergent members.

### Evolution of *BnMS5* alleles and closely related homologs

The phylogenetic analysis indicates that *BnMS5* belongs to a lineage (Lineage‐12 in Fig. S3) that contains 14 homologs (gene loci) from eight species in Clade B (10 from five *Brassica* species, three from two *Raphanus* species, one from *Sisymbrium irio*) and one from *Arabis alpina* in Clade D (Table S4). As the *MS5* alleles in *B. napus* have distinct functions, we investigated the evolution of the *MS5* alleles of *Brassica* species, by identifying the genomic synteny regions in nine Brassicaceae species and two outgroup species, using the eight flanking genes of *Bra018456* (*A08003827*, the *BrMS5^c^* allele) in *B. rapa* (Fig. S5). The results show clear synteny blocks in the genomes of *A. thaliana* (from *At1g10120* to *At1g10220*) and other species, with *MS5* homologs present in the syntenic regions of the two *Brassica* basic diploids (*B. rapa* ChrA5 (*Bra018456*) or *B. oleracea* ChrC8 (*Bol022070*)). Nevertheless, the flanking genes adjacent to *BnMS5* (e.g. *BnaA08g25980D*, *BnaA08g25970D*, *BnaA08g25900D* and *BnaA08g25890D*) correspond to *A. alpina* genes that are located in Chr1, but this region does not contain a homolog of *BnMS5*. The syntenic region of the *BnMS5* flanking genes in *S. irio* also is located in a different genomic region from that of the *MS5* homolog in *S. irio*. The closest homolog of *BnMS5* in *S. irio* (*scaffold689_101*) is located in another genomic region. Moreover, the adjacent genomic regions of *A. alpina*_*KFK32194.1* and *S. irio*_*scaffold689_101* exhibit a weak syntenic relationship. Thus, *MS5* probably originated in the common ancestor of clades B (*Brassica*) and D (*A. alpina*), and then was relocated to the current genomic region before the divergence of *B. rapa* and *B. oleracea*; however, *BnMS5* homologs in Lineage‐12 might have been lost in *A. thaliana* (and other species in Clade A), as well as in *E. salsugenium* and *S. parvula* in Clade B.

**Fig. 3:**
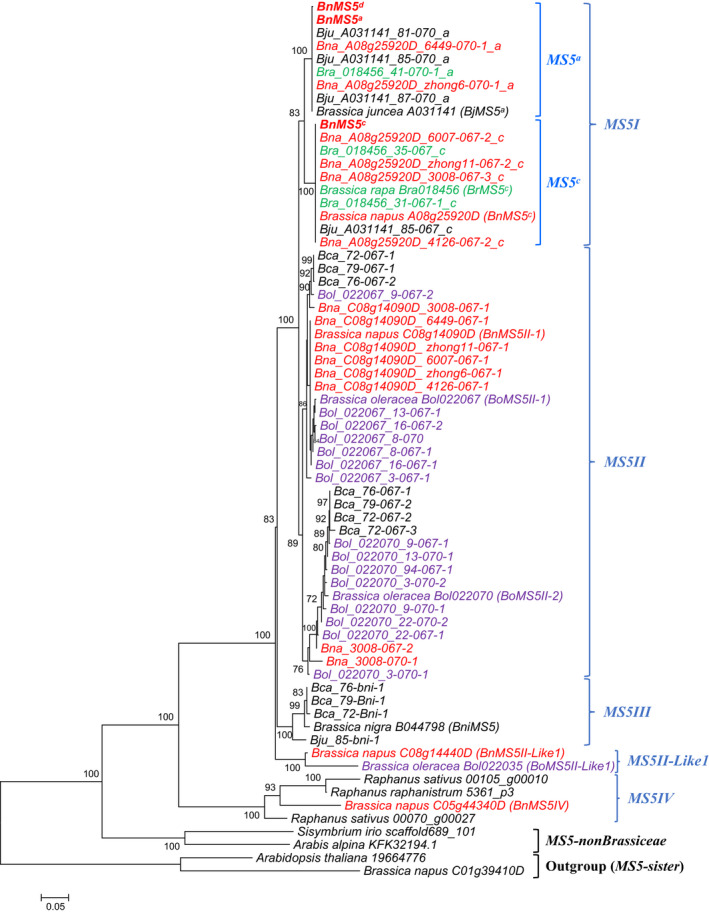
Maximum‐likelihood tree that illustrates the phylogenetic relationships of *MALE STERILITY 5* (*MS5*) closely related homologs in Brassicaceae. Gene IDs with species full names represent sequences identified in public genomic data, and the others represent sequences cloned in this study (see the Materials and Methods section). Here, for convenience, we use *MS5I* and *MS5II* to distinguish different *Brassica napus (Bn)MS5* homologs that are derived from either *Brassica rapa* or *B. oleracea*. Red, sequences from *B. napus*; green, *B. rapa*; and purple, *B. oleracea*. Bootstrap values (≥ 70) are shown for each node. Bra, *B. rapa*; Bol, *B. oleracea*; Bca, *B. carinata*; Bni, *B. nigra*; Bju, *B. juncea*.

In order to compare closely related *BnMS5* homologs from *Brassica* species, including diploids with the AA, BB and CC genomes, as well as the three allotetraploids *B. juncea* (AABB), *B. napus* (AACC) and *B. carinata* (BBCC), we designed primers using sequences of 10 *MS5* homologs to identify full‐length CDS of 47 *MS5* homologs and alleles (Fig. S6; Table S8; Dataset S3). Phylogenetic analyses grouped these sequences into several clades consistent with speciation and gene duplication events (Fig. 3). First, the *MS5I* clade consists of the *MS5^a^* and *MS5^c^* subclades, both with *B. rapa* homologs and alleles, and diverged after the speciation of *B. rapa* and before the emergence of *B. napus*. Second, the *MS5II* clade consists of homologs and alleles derived from *B. oleracea*, which are clustered into two subclades indicating a gene duplication in *B. oleracea*. Third, the *MS5III* clade consists of *B. nigra* homologs and alleles, as a sister clade of the *MS5I* + *MS5II* clade. Thus, *MS5I*, *MS5II* and *MS5III* likely resulted from the speciation events of the three basic diploids. Fourth, the combined clade of *MS5I*, *MS5II* and *MS5III* is sister to *MS5II‐Like1*, which contains only two homologs from *B. oleracea* and *B. napus*, respectively. Nevertheless, the chromosomal positions of *BoMS5II‐Like1* is near its intraspecific homologs in the *MS5II* clade, suggesting that *BoMS5II‐Like1* and *BoMS5II‐1/2* might have been generated from duplication occurred before the divergence of *B. rapa*, *B. oleracea* and *B. nigra*, and then was inherited in *B. napus*. Finally, *MS5IV* consists of three homologs (gene loci) from *Raphanus* species and one homolog (gene locus) from *B. napus* and is sister to the clade with all above‐mentioned genes.

Within the *MS5^c^* subclade, several sequences of the *BnMS5^c^* allele from *B. napus* maintainer lines included here displayed 100% CDS sequence identity with sequences from the *B. rapa* accessions Bra35 and Bra31 (Fig. 3). Likewise, homologs of the *BnMS5^a^* allele from selected restorer lines showed 100% CDS sequence identity with homologs from *B. rapa* accession Bra41 and *B. juncea* accessions, and were in the *MS5^a^* subclade. *BnMS5^d^*, which has a single C/T transition (mis‐sense mutation: L/F) compared with *BnMS5^a^*, also was in the *MS5^a^* clade. In addition, *B. rapa* accessions carried either *MS5^a^* or *MS5^c^*, indicating that *BnMS5^a^* of the *B. napus* restorer lines was probably derived from *B. rapa* accession(s) carrying *MS5^a^*, whereas *BnMS5^c^* of the *B. napus* maintainer lines was derived from *B. rapa* accession(s) carrying *MS5^c^*.

Taken together, we summarize the evolution of *BnMS5* briefly here. Because *B. napus* is a hybrid of *B. rapa* and *B. oleracea*, two *MS5* loci are found in *B. napus*. One of them is ‘*BnA08g25920D* (*BnMS5*)’ (orthologous to *Bra018456* in ChrA5 of *B. rapa*), whereas the other is ‘*BnC08g14090D* (*BnMS5II‐1*)’ (orthologous to *Bol022070* on ChrC8 in *B. oleracea*). In *B. napus*, *BnA08g25920D* (*BnMS5*) has three natural alleles; *BnMS5^a^*, *BnMS5^c^* and *BnMS5^d^*. The *MS5^a^* and *MS5^c^* alleles were generated before the divergence of *B. rapa* and *B. napus*, and *B. napus* had inherited both alleles from *B. rapa* through hybridization. The *BnMS5^d^* allele then was created by a single nucleotide substitution in *B. napus*.

### *MS5* homologs show similar expression patterns and variable protein sequences

Frequencies of the *BnMS5^a^MS5^a^* and *BnMS5^c^MS5^c^* genotypes were investigated in 50 *B. napus* inbred lines from different areas by scoring the fertility of F_1_ hybrids between an inbred line and the sterile line (*BnMS5^d^MS5^d^*). The genotype frequency of maintainer line (*BnMS5^c^MS5^c^*, 82%) was significantly higher than the restorer line (*BnMS5^a^MS5^a^*, 18%), without detection of the sterile genotype *BnMS5^d^MS5^d^* (Fig. S7; Table S1). Previous analysis showed a similar genotype frequency of *BnMS5^c^MS5^c^* (81.7%) in 186 inbred lines of *B. napus* (Xin, 2016). Sequence analysis of 30 *B. rapa* varieties (Table S2) indicated that 47% were *BrMS5^a^MS5^a^* and 53% *BrMS5^c^MS5^c^*. These results suggest that the frequencies of the *MS5^a^* and *MS5^c^* genotypes are different between *B. rapa* and *B. napus*. Previous analyses showed that *BnMS5^a^* and *BnMS5^c^* were expressed in various organs with different expression patterns (Xin *et al*., 2016; Zeng *et al*., 2016). Here we analyzed the expression patterns of *BrMS5^c^* (*Bra018456*), *BrMS5^a^*, *BoMS5II‐1* (*Bol13‐067‐1*) and *BoMS5II‐2* (*Bol9‐070‐1*) in basic diploids, and found that these *MS5* alleles and homologs also are expressed in various organs (Fig. S8a). In addition, we analyzed the activity of three promoters, the *B. rapa*‐derived Pro*BnMS5^a^*, Pro*BnMS5^c^* and the *B. oleracea*‐derived Pro*BnMS5II*, using GUS fusions, showing expression in multiple organs of transformed *A. thaliana* (Fig. S8b). The results of promoter analysis indicated that the expression patterns of *BnMS5^a^*, *BnMS5^c^* and their closely related *MS5* homologs are similar. Moreover, it is demonstrated that the male fertility‐associated function of *BnMS5* alleles/homologs in *B. napus* depended on the variations in protein sequences more than changes in expression (Xin *et al*., 2020).

**Fig. 4:**
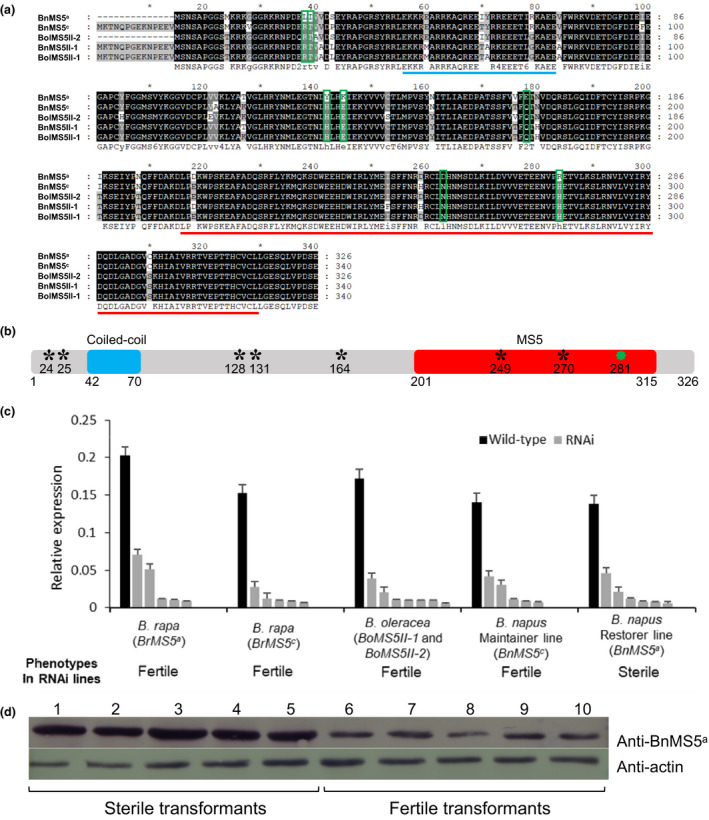
Molecular and functional characterization of *MALE STERILITY 5* (*MS5*) homologs. (a) Sequence alignment and domains of *Brassica napus* (Bn)MS5 protein homologs. The conserved domains are marked with lines below the sequences: blue line, the coiled‐coil domain; red line, the MS5 domain (formerly named as DUF626). Rectangular boxes indicate seven specific amino acid substitutions between BnMS5^a^ and others. (b) An illustration of the BnMS5^a^ protein and its domains. The conserved domains are shown as boxes: blue box, the coiled‐coil domain; red box, the MS5 domain. Black asterisks indicate seven specific amino acid substitutions between BnMS5^a^ and the other four proteins. The green asterisk indicates one amino acid substitution between BnMS5^a^ and BnMS5^d^. (c) Transcript level of *MS5* homologs and phenotypes in RNAi lines. Expression of *MS5* homologs in the inflorescences of independent RNAi T_0_ lines are relative to the corresponding wild‐type (WT) plants using quantitative real‐time PCR. Error bars represent SD. The mRNA level of *MS5* homologs in each RNAi line was significantly lower than in the WT control (*P* < 0.05). (d) Western blot analysis of the BnMS5 protein in the Zhong6 background (*BnMS5^a^MS5^a^*) that carries *BnMS5^d‐trans^*. The upper bands represent the BnMS5 signals. The lower bands represent the *B. napus* actin signal. Lanes 1–5, proteins from sterile transformants. Lanes 6–10, proteins from fertile transformants.

In order to analyze protein domains and sequence variation among alleles and closely related homologs of BnMS5^a^, we selected protein sequences of BnMS5^a^, BnMS5^c^, BnMS5II, BoMS5II‐1 and BoMS5II‐2 (Fig. 4a). Sequence alignment of the five MS5 proteins showed high levels of sequence similarity (> 90%), with a total of only 38 aa alterations. In addition, the BnMS5^a^ and BoMS5II‐2 proteins lack the first 14 aa found in the other three proteins, with additional aa alterations (Fig. 4a). Two conserved domains were identified: a coiled‐coil domain, at aa42‐70 in BnMS5^a^ and BoMS5II‐2, and aa56‐84 in BnMS5^c^, BoMS5II‐1 and BnMS5II, and the MS5 domain in the C‐terminal regions (aa201‐315 in BnMS5^a^ and BoMS5II‐2, and aa215‐329 in BnMS5^c^, BoMS5II‐1 and BnMS5II). Interestingly, compared with BnMS5^a^, BnMS5^d^ has one amino acid substitution (L281F) that is not among the aa variation sites between BnMS5^a^ and BnMS5^c^ (Fig. 4b), suggesting that this difference between BnMS5^a^ and BnMS5^d^ might contribute to their functional difference.

### The *MS5^a^* allele has gained a novel male fertility‐related function probably since the divergence of *B. rapa* and *B. oleracea*

In order to investigate the functions of *MS5* alleles and homologs from *Brassica* species, we introduced various cDNA constructs into the *B. napus* male‐sterile line TE5A (*BnMS5^d^MS5^d^*). Six recombinant vectors were generated containing native promoter and full CDSs (primers shown in Table S8) of *B. napus BnMS5II‐1*, *B. rapa BrMS5^a^* (*Bra41‐070‐1*) and *BrMS5^c^* (*Bra018456*), *B. oleracea BoMS5II‐1* (*Bol13‐067‐1*) and *BoMS5II‐2* (*Bol9‐070‐1*), and *B. nigra BniMS5* (*BniB044798*), and then transformed into the TE5A line. We found that seven of nine T_0_ TE5A transgenic plants with the *BrMS5^a^* construct were fertile, but the transgenic plants with any of the other five constructs were sterile (Table S9). The restored fertility co‐segregated with the *BrMS5^a^* transgene in the segregating transgenic T_1_ progeny population. Therefore, only *MS5^a^* (including *BnMS5^a^* and *BrMS5^a^*) was able to confer male fertility in *B. napus*.When the constructs containing the entire coding region of *BnMS5^c^*, *BoMS5II‐1* or *BoMS5II‐2* fused to the respective native promoters were introduced into the male‐sterile (*MS5^b^MS5^c^*) plants, the transgenic plants with any of the constructs were sterile (Xin, 2016; Xin *et al*., 2020). Furthermore, when the entire coding region of *BnMS5^c^*, *BoMS5II‐1* or *BoMS5II‐2* fused to the *BnMS5^a^* promoter were introduced into the male‐sterile (*MS5^b^MS5^c^*) plants, respectively, some of the transgenic plants with *MS5^c^* showed restored fertility, but all of the transgenic plants with *BoMS5II‐1* or *BoMS5II‐2* remained complete male‐sterile (Xin, 2016; Xin *et al*., 2020). These results revealed that the *BnMS5^c^* allele contributes to fertility in *B. napus*, but the *MS5II‐1* and *MS5II‐*2 genes could not rescue the defects of *MS5^b^* in male fertility.

In order to further test the functions of *Brassica MS5* homologs, RNA interference (RNAi) was used to reduce gene expression. Because *Brassica MS5* clade members shared > 90% sequence similarity, a 400‐bp fragment starting from the ATG initial codon of the *BnMS5^c^* cDNA sequence was selected to construct the RNAi binary vector, which was transformed into *B. rapa* lines that carried either *BrMS5^a^MS5^a^* or *BrMS5^c^MS5^c^*, *B. oleracea*, the *B. napus* maintainer line (Zhong11 with *BnMS5^c^MS5^c^*) and *B. napus* restorer line (6449 with *BnMS5^a^MS5^a^*). The qRT‐PCR analyses of inflorescences showed a significant reduction of the expression of *MS5* homolog in the five kinds of transgenic lines compared to the WT plants (Fig. 4c). Notably, six knockdown transgenic T_0_ plants in *B. napus* restorer line (*BnMS5^a^MS5^a^*) showed complete male sterility, whereas all other *MS5* knockdown transgenic plants, in the backgrounds of *B. rapa*, *B. oleracea* and *BnMS5^c^MS5^c^* maintainer lines, exhibited normal fertility (Fig. 4c). These results indicated that the function of *MS5^a^* is required for male fertility in *B. napus*. Taken together, the results collectively revealed that *MS5^a^* allele has gained a novel male fertility‐related function that is essential in *B. napus*, probably since the origin of *B. rapa*.

### A *BnMS5^d^* transgene can inhibit *BnMS5^a^* function in a dosage‐dependent manner

We isolated the complete genomic fragment of *BnMS5^d^*, including *c*. 1000 bp of the putative promoter region and cloned it into the pCAMBIA2300 binary vector, and designated this as 2300‐MS5^d^. Interestingly, when the resulting construct 2300‐MS5^d^ was transformed into a restorer line with *BnMS5^a^MS5^a^*, some transgenic plants were male sterile (five of 10 transgenic plants). This suggested that the *BnMS5^d^* transgene (designated as *BnMS5^d‐trans^*) could inhibit the function of *BnMS5^a^* in the restorer line, in contrast to the previous genetic result of *BnMS5^a^* being dominant over *BnMS5^d^* (Fig. S1).

It is possible that *BnMS5^d‐trans^* was expressed relatively highly compared with that of *BnMS5^a^*. Transgenes are known to have varying copy number, which can affect the expression levels (Yi *et al*., 2008; Gadaleta *et al*., 2011). To estimate the copy number of *BnMS5^d‐trans^*, we used duplex droplet digital PCR (ddPCR) (Glowacka *et al*., 2016; Xu *et al*., 2016; Collier *et al*., 2017), with CruA (four copies for ddPCR) in *B. napus* as a reference (Wu *et al*., 2010). The *BnMS5^d‐trans^* transgene copy number of positive transformants was estimated according to the ratio of *NPTII/CruA* and *P35S/CruA* by duplex ddPCR. The ddPCR detected 6–48 copies of *BnMS5^d‐trans^* in sterile transgenic plants, but only 1–4 copies of *BnMS5^d‐trans^* in fertile transformants (Table S13). When the copy number ratio (*BnMS5^d‐trans^*/*BnMS5^a^*) was ≤ 4, the transgenic plants were fertile, indicating that *BnMS5^d‐trans^* was unable to inhibit *BnMS5^a^*. When the copy number ratio (*BnMS5^d‐trans^*/*BnMS5^a^*) was > 4, the transgenic plants were sterile, meaning that *BnMS5^d‐trans^* could inhibit *BnMS5^a^*. Furthermore, the accumulation of BnMS5 protein in sterile transformants was higher than that in fertile transformants (Fig. 4d), indicating that sterility was not due to co‐suppression mediated by a high copy number of the transgene.

### *BnMS5^a^* and *BnMS5^d^* show different nuclear envelope‐related dynamics during early meiosis in *B. napus*

**Fig. 5:**
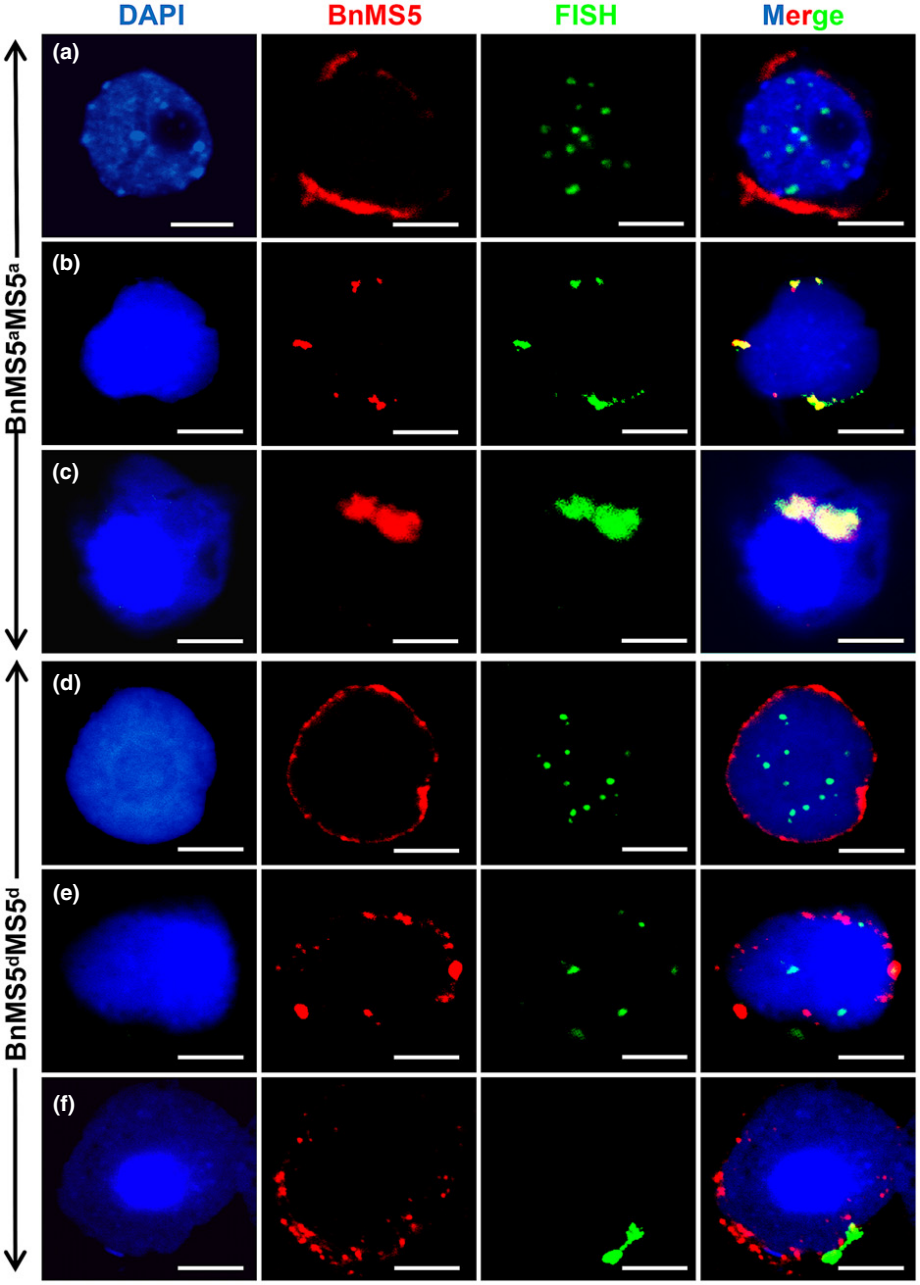
Subcellular localization of BnMS5^a^ and BnMS5^d^ relative to telomeres during early meiosis (Bn, *Brassica napus*; MS5, MALE *STERILITY 5*). Immunolocalization using rabbit polyclonal antibody against MS5 proteins (red) in plants carrying the *BnMS5^a^* or *BnMS5^d^* alleles and fluorescence in situ hybridization using a 2.6‐kb direct repeat of telomeric sequence in pWY86 (green). Chromosome DNA was counterstained with 4′,6‐diamidino‐2‐phenylindole (blue). Merged images show the overlap of green, red and blue fluorescence. Early leptotene (a) and (d), late leptotene (b) and (e), and early pachytene (c) and (f). Bars, 10 μm.

Previous study suggested that *BnMS5^a^* might be involved in meiotic telomeric movement (Xin *et al*., 2016). To test this hypothesis, double‐immunolabeling of BnMS5 protein and telomeres was performed using > 50 male meiocytes of WT *BnMS5^a^MS5^a^* and TE5A mutant *BnMS5^d^MS5^d^*, respectively. The cells at different meiotic stages were determined by the telomere FISH signals and chromosome morphology (Hamant *et al*., 2006). At early leptotene phase, BnMS5^a^ and BnMS5^d^ were distributed around the nuclear envelope (NE) in *BnMS5^a^MS5^a^* and *BnMS5^d^MS5^d^*, respectively, whereas telomere FISH signals were dispersedly localized in the nucleus (Fig. 5a,d). At late leptotene, BnMS5^a^ and BnMS5^d^ formed aggregates of variable sizes at the NE (Fig. 5b,e), respectively, in *BnMS5^a^MS5^a^* and *BnMS5^d^MS5^d^*. Simultaneously, telomeres were clustered and colocalized with the BnMS5^a^ aggregates (Fig. 5b), whereas telomere clustering *BnMS5^d^MS5^d^* did not overlap with the BnMS5^d^ aggregates (Fig. 5e). At early pachytene, telomeres co‐localized with BnMS5^a^ in larger foci in the NE in *BnMS5^a^MS5^a^* (Fig. 5c), but the large telomeres foci in *BnMS5^d^MS5^d^* did not co‐localize with the BnMS5^d^ aggregates, which remained relatively small (Fig. 5f). In short, BnMS5^a^ aggregated and co‐localized with telomeric clusters at early pachytene, but BnMS5^d^ could not aggregate, even though telomeres are still able to cluster. In addition, we examined more than 30 meiocytes from *BnMS5^c^MS5^c^* and *BrMS5^a^MS5^a^* by immunolabeling of BnMS5 protein. The immunostaining signals were detected in NE of meiocytes in *BnMS5^c^MS5^c^* and *BrMS5^a^MS5^a^* (Fig. S9).

### Interaction between BnMS5 and SUN‐domain proteins

**Fig. 6:**
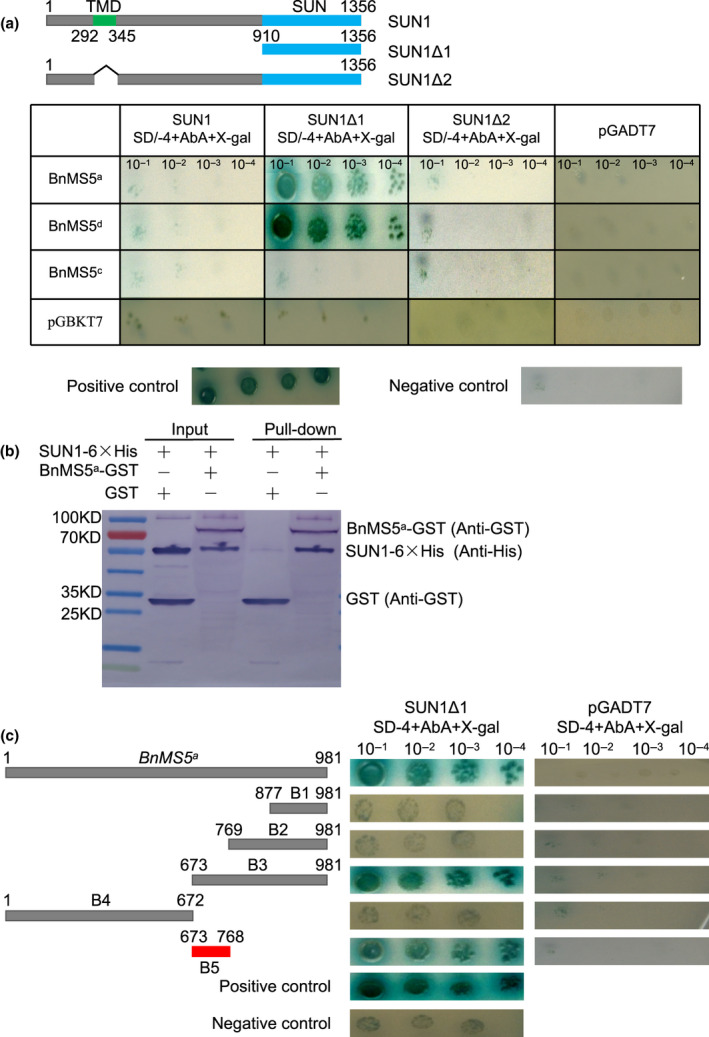
Interaction between allelic BnMS5 forms and nuclear envelope protein SUN1 (Bn, *Brassica napus*; MS5, MALE STERILITY 5). (a) BnMS5^a^, BnMS5^d^ and BnMS5^c^ were constructed into the pGBKT7 plasmid as baits, respectively. SUN1, SUN1Δ1 and SUN1Δ2 were cloned into the pGADT7 vector as preys, respectively. Yeast two‐hybrid (Y2H) assays showed that BnMS5^a^ and BnMS5^d^ interacted with the SUN domain of SUN1. Positive control, co‐transformation of positive plasmids pGBKT7‐p53 and pGADT7‐RecT; negative control, co‐transformation of negative plasmids pGBKT7‐Lam and pGADT7‐RecT. (b) Physical interaction of BnMS5^a^ and SUN1 *in vitro* detected using a Glutathione S‐Transferase (GST) pull‐down assay. BnMS5^a^‐GST was incubated in binding buffer containing glutathione‐agarose beads with or without SUN1‐6 × His, and agarose beads were washed for five times and eluted. Lysis of *Escherichia coli* (Input) and eluted proteins (Pull‐down) from beads was immunoblotted using anti‐HIS and anti‐GST antibodies. The marker was PageRuler™ Prestained Protein Ladder. (c) A series of truncated BnMS5^a^ mutants B1, B2, B3, B4 and B5 were constructed into the pGBKT7 plasmid as baits, respectively. Determination of the interaction region between BnMS5^a^ and SUN1Δ1. Positive control, co‐transformation of positive plasmids pGBKT7‐p53 and pGADT7‐RecT; negative control, co‐transformation of negative plasmids pGBKT7‐Lam and pGADT7‐RecT.

SUN (Sad‐1/UNC‐84) domain proteins play important roles in linking telomeres to NE during meiosis (Ding *et al*., 2007). Because BnMS5 is situated at the NE, we tested whether BnMS5 could interact with SUN proteins. SUN proteins typically have an N‐terminal and a C‐terminal regions, which are separated by one or more transmembrane domains (TMDs) (Tzur *et al*., 2006). We tested different portions of SUN1 from *B. napus*, including the full‐length SUN1 (SUN1), a fragment containing only SUN domains (SUN1Δ1), and a truncated protein without the TMD domain (SUN1Δ2) (Fig. 6a). Western blots with Anti‐Gal4BD or Anti‐Gal4AD showed that both bait and prey fusions proteins were expressed in yeast cells (Fig. S10). Y2H experiments showed that BnMS5^a^ and BnMS5^d^ interacted with the SUN1‐SUN domain, respectively, whereas BnMS5^c^ did not (Fig. 6a). As negative Y2H result does not necessarily mean that MS5^c^ and SUN do not interact in *B.napus*. Therefore further experiments, such as BiFC, could be more informative about the interaction between MS5^c^ and SUN in *B. napus*. Physical interaction between BnMS5^a^ and SUN1 *in vitro* also was observed in the GST pull‐down assays (Fig. 6b).

In order to determine the roles of different BnMS5^a^ regions in the interaction with SUN1Δ1, sequential deletion mutants of *BnMS5^a^* (B1, B2, B3, B4 and B5) were generated (Fig. 6c). Y2H assays showed that truncated proteins lacking amino acids 1 to 293 (B1) or 1 to 256 (B2) of BnMS5^a^, and that lacking from aa325 to 309 (B4) failed to interact with the SUN1‐SUN domain, whereas both the BnMS5^a^ protein lacking aa1 to 224 (B3) and the fragment from aa225 to 256 (B5) fully interacted with the SUN1‐SUN domain (Fig. 6c). These results showed that the BnMS5^a^ domain with aa225 to 256 allowed interaction with the SUN domain of SUN1. BnMS5^d^ also was able to interact with the SUN1‐SUN domain (Fig. 6a), consistent with the fact that the mutation site of *BnMS5^d^* is at the 841 bp site, outside the region (673–769 bp) of the domain in BnMS5^a^ for the interaction with SUN1.

## Discussion

### Evolutionary history of *BnMS5* and its alleles

**Fig. 7:**
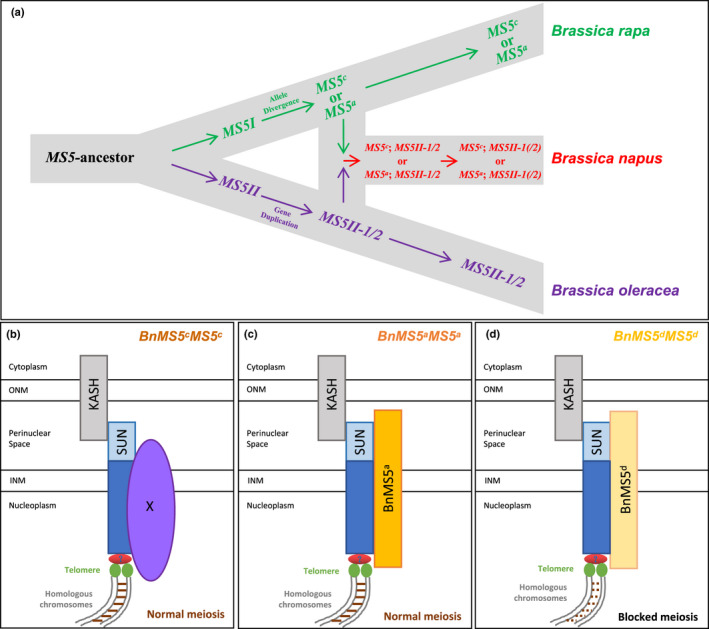
Evolutionary history of *Brassica napus MALE STERILITY 5* (*BnMS5*) and a potential model underlying the establishment of different functions by *BnMS5* alleles. (a) A summary of the evolutionary history of *MS5* homologs and different *MS5* alleles in *Brassica rapa*, *B. oleracea* and *B. napus* according to Fig. 3. Background gray blocks indicate the species relationships of these three species, showing that *B. napus* is derived from hybridization between the ancestors of other two species. *MS5*‐ancestor indicates the ancestral *MS5* gene in the last common ancestor (LCA) of *B. rapa* and *B. oleracea*. *MS5I* and *MS5II* indicate *MS5* homologs in *B. rapa* and *B. oleracea*, respectively, after the divergence of the two species. Before the hybridization between *B. rapa* and *B. oleracea*, both *MS5^a^* and *MS5^c^* alleles had already existed in *B. rapa*, and *MS5II* in *B. oleracea* had likely already experienced a duplication event resulting in two copies of *MS5II* (*MS5II‐1* and *MS5II‐2*). Therefore, some of the direct *B. napus* lines have *MS5^c^* plus *MS5II‐1* and *MS5II‐2*, whereas the others have *MS5^a^* plus *MS5II‐1* and *MS5II‐2* homologs. After the hybridization, however, *BnMS5II‐2* homologs likely experienced gene loss events in some *B. napus* plants, so that some of the extant *B. napus* plants still have both *BnMS5II‐1* and *BnMS5II‐2* homologs, whereas the others retain one homolog. (b–d) A potential working model of three allelic BnMS5 forms. ONM, outer nuclear membrane; INM, inner nuclear membranes; KASH, Klarsicht/ANC‐1/Syne homology. (b) BnMS5^c^ does not interact with the nuclear envelope SUN protein and an unidentified protein X is responsible for meiotic chromosomal behavior through interaction with SUN proteins in *BnMS5^c^MS5^c^*. (c) BnMS5^a^–SUN proteins complex is involved in fertility control in *BnMS5^a^MS5^a^*. (d) The single‐nucleotide C‐T transition (missense mutation: L/F) in *BnMS5^a^* affected its normal function in facilitating meiosis, which, in turn, resulted in male sterility in *BnMS5^d^MS5^d^*. BnMS5^a^ and BnMS5^d^ are in a functionally antagonistic state, performing functions in a dose‐dependent or competitive manner.

Gene duplication is one of the major routes for the birth of new genes (Kaessmann, 2010). Based on the phylogenetic relationships of *MALE STERILITY 5* (*MS5*) homologs and different *MS5* alleles in closely related Brassicaceae species, we concluded the evolutionary history of *MS5* homologs and different *MS5* alleles in three *Brassica* species (Fig. 7a). It was likely that the *B. napus BnMS5* clade arose from the duplication of a MS5 domain‐containing gene in the ancestor of Brassicaceae, potentially followed by translocations and a particularly rapid evolution. Before the hybridization between *B. rapa* and *B. oleracea* happened, at least two alleles (*MS5^a^* and *MS5^c^*) had already existed in *B. rapa*; meanwhile, *MS5II* in *B. oleracea* had already experienced a duplication event resulting in two copies of *MS5II* (*MS5II‐1* and *MS5II‐2*). Therefore, the direct *B. napus* offspring had either *MS5^c^* allele and *MS5II‐1/2*, or *MS5^a^* allele and *MS5II‐1/2*. After hybridization, however, *BnMS5II‐2* homologs likely experienced gene loss events in some *B. napus* plants, so that some of the extant *B. napus* plants still have both *BnMS5II‐1/2* homologs whereas the other ones retain one homolog.

### Differential requirements for function of *BnMS5^a^* and *BrMS5^a^*

A gene is considered to be essential to an organism when the loss of its function affects fitness of the organism; otherwise, the gene is said to be nonessential (Chen *et al*., 2010). Our studies suggest that different *MS5* homologs and alleles were initially nonessential or redundant for the development and survival of basic *Brassica* diploids. Then, in the genetic context of *B. napus*, *MS5^a^* subsequently became indispensable for male reproductive development. Although the *BnMS5^a^* and *BrMS5^a^* alleles showed 100% CDS sequence identity and the same expression pattern (Fig. S9), an RNAi experiment of the *BrMS5^a^* (*Bra018456*) allele did not reduce fertility, suggesting that *MS5^a^* is more important for male fertility in *B. napus* than in *B. rapa*. The male fertility‐associated function of *BnMS5* alleles/homologs depended on the dimer of BnMS5 that are likely dosage‐dependent in *B. napus* (Xin *et al*., 2020); therefore, it is possible that low dosage MS5^a^ still could play a role in fertility of *B. rapa*. If this is the case, then the new function in *MS5^a^* could have evolved before the origin of *B. napus*. Furthermore, because the close homologs of *MS5* in *B. oleracea* (*Bol022067* and *Bol022070*) do not seem to have the *MS5^a^* function, the new function probably evolved after the divergence of *B. rapa* and *B. oleracea* (~4.6 Myr ago).

### *MS5^a^* and *MS5^c^* conferred distinct function of male‐fertile in *B. napus*

New genes could acquire novel functions via adaptive evolution (Chen *et al*., 2013). Reproduction‐related genes are highly divergent and more rapidly evolving than the other genes of a particular species (Swanson & Vacquier, 2002). Although most of the *Brassica MS5‐Like* family members were highly similar in DNA sequence, the *MS5* knockdown results showed that only *MS5^a^* display a novel male fertility‐associated function, which is required for male fertility in *B. napus*. Nevertheless, a recent study using CRISPR/Cas9 confirmed that the *MS5^c^* allele also was necessary for male fertility, but it could not restore male fertility in *MS5^b^MS5^c^* hybrids of *B. napus* (Xin *et al*., 2020). Further research revealed that the functional divergence of the male‐fertile alleles *MS5^a^* and *MS5^c^*, was strongly dependent on the variations in the coding sequences, although the differences in expression levels possibly due to the variations in promoter sequence also played a lesser role (Xin *et al*., 2020). The observations that both *MS5^a^* and *MS5^c^* have the male fertility‐associated function might have suggested that the genotype frequencies of *MS5^a^* and *MS5^c^* should be similar in *B. napus*. Intraspecies analysis of *MS5* genotype frequencies showed that *MS5^a^* allele has a lower frequency than *MS5^c^* in *B. napus*, whereas the genotype frequencies of *MS5^a^* and *MS5^c^* were equal in *B. rapa*. The A subgenome of most *B. napus* varieties is thought to have evolved from the ancestor of European turnip (Lu et al., 2019); therefore, the difference between the two species in frequencies of *MS5^a^* and *MS5^c^* might be caused by initial sampling effect, leading to an over‐representation of the *MS5^c^* allele in the ancestral population of *B. napus*.

### Integration of *BnMS5^a^* into existing genetic networks

Following the acquisition of novel functions, new genes play essential roles in developmental and reproductive processes by becoming rapidly integrated into pre‐existing interaction networks (Chen *et al*., 2010). Our studies showed that BnMS5^a^ and BnMS5^d^ are both able to interact with the nuclear envelope protein SUN1; also, BnMS5^a^ and telomeres have the same dynamics in restorer lines. However, BnMS5^d^ has abnormal movements in the nuclear envelope in spite of the normal telomeric dynamics of the meiocytes expressing BnMS5^d^, leading to male sterility. Because BnMS5^a^ is involved in establishment of meiosis‐specific chromosome structure during early prophase I, including homologous recombination, installation of SYN1 and central element, it is reasonable to speculate that synergistic movement of BnMS5^a^ and telomeres together with additional components contribute to proper meiotic chromosome structure and movements (Xin *et al*., 2016). These findings suggested that there might be other interaction partners of MS5 that are involved in this network, and these unknown proteins might form a complex with BnMS5^a^ and SUN1 to regulate the meiotic behavior of homologous chromosomes. In the absence of BnMS5^a^, BnMS5^d^ causes male sterility probably due to its failure to interact properly with such additional partners, whereas in the presence of sufficient BnMS5^a^ (relative to BnMS5^d^), the amount of functional BnMS5^a^/SUN proteins was enough to ensure fertility.

### A model for the establishment of different functions of *BnMS5* alleles

We found that BnMS5^c^ from the maintainer lines failed to interact with SUN1, suggesting that the regulatory network of *BnMS5^c^* may be different from that of *BnMS5^a^*. Because *BnMS5^c^* is necessary for male fertility as a homodimer (Xin *et al*., 2020), we deduce that unlike BnMS5^a^ which is able to interact with SUN1 for male fertility, BnMS5^c^ possibly has not evolved a similar function as BnMS5^a^ in the maintainer lines, but could alternatively interact with another meiosis‐related protein to affect male fertility. Alternatively, in the maintainer lines, another pathway or gene (designated as X) could be responsible for fertility control through interaction with SUN proteins (Fig. 7b). *BnMS5^d^* was able to cause sterility when introduced into the *BnMS5^c^MS5^c^* maintainer line (zhong11) (Zeng *et al*., 2016), suggesting that interaction between BnMS5^d^ and SUN proteins could reduce the interaction of X and SUN. In addition, BnMS5^a^ and BnMS5^d^ proteins could both interact with SUN proteins *in vitro*, which suggested that BnMS5–SUN complex could result in their corresponding traits: the BnMS5^a^–SUN complex was involved in fertility control (Fig. 7c); whereas the single amino acid substitution in BnMS5^d^ affected its function somehow, resulting in male sterility (Fig. 7d). Moreover, BnMS5^a^ and BnMS5^d^ both interacted with the SUN1‐SUN domain, providing a mechanism for their mutual inhibition, in a dose‐dependent or competitive manner. For hybrids between *BnMS5^a^MS5^a^* and *BnMS5^d^MS5^d^*, although only half of the normal amount of *BnMS5^a^* gene products was generated, this was sufficient in maintaining the normal phenotype in the *BnMS5^a^MS5^d^* hybrids because the ratio of *BnMS5^d^*/*BnMS5^a^* = 1. The results support the idea that BnMS5^d^ retained domains capable of interacting with SUN proteins, which led to directly antagonizing the action of the full‐length BnMS5^a^, and also challenged the assumption that the function of *BnMS5^a^* might require a threshold level of transcripts.

## Author contributions

XZ, HL, HM, XY and GW initiated, conceived and supervised the study and wrote the manuscript; HL performed evolutionary analyses; KL, RY, SZ and J. Luo performed experiments and analyzed data; XL and J. Li provided the technical assistance; and all authors read and approved the manuscript. XZ and HL contributed equally to this work.

## Supporting information


**Dataset S1** Protein sequences of 727 *MS5‐Like* homologs identified in this study.

**Dataset S2** Alignment of 727 *MS5‐Like* CDS sequences used for phylogenetic inferences in Fig. S3.

**Dataset S3** Full‐length CDS homologs of *MS5* isolated from 22 different *Brassica* species.

**Fig. S1** Classical genetic model and three‐line hybrid breeding procedure of the genic male sterile system TE5ABC in *B. napus*.
**Fig. S2** Phylogenetic relationships of 23 Brassicaceae species belonging to four clades based on a published Brassicaceae phylogeny (Huang *et al.*, 2016).
**Fig. S3** Maximum‐likelihood tree of *MS5‐Like* family inferred using 727 homologs which are divided into 25 homolog lineages (bootstrap values ≥60).
**Fig. S4** Maximum‐likelihood tree of *MS5‐Like* gene family inferred using 701 homologs (length ≥100 aa) which could be also divided into 25 homolog lineages (bootstrap values ≥60) as Fig. S3.
**Fig. S5** Synteny of the *MS5* locus‐related genomic regions in nine Brassicaceae genomes and two outgroup species.
**Fig. S6** Nucleotide sequence alignment of 10 *MS5* homologs/alleles and primers.
**Fig. S7** Gene frequencies of the *MS5* locus in populations of *B. napus* and *B. rapa*.
**Fig. S8** Expression patterns and promoter activity of *MS5* homologs.
**Fig. S9** Subcellular localization of BnMS5^c^ and BrMS5^a^ during early leptotene meiosis.
**Fig. S10** Western blot of bait or prey fusion proteins in yeast cells.

**Table S1**The information for 50 *B. napus* inbred lines.
**Table S2** The information for 30 diverse *B. rapa* accessions.
**Table S3** The information for 22 diverse *Brassica* species accessions.
**Table S4** List of 83 organisms and their *MS5* homolog numbers.
**Table S5** 727 *MS5‐Like* homologs in 23 Brassicaceae species and their protein lengths.
**Table S6** *MS5* homologs from 8 Brassicaceae species in 25 phylogenetic groups and their syntenic relationships.
**Table S7** Pairwise syntenic homologs from 8 Brassicaceae species in 25 phylogenetic groups.
**Table S8** DNA oligonucleotide sequences.
**Table S9** Results of the genetic complementation analysis of the TE5A mutant (*BnMS5^d^MS5^d^*) with six *MS5* homologs/alleles.
**Table S10** Number of homologs in 25 phylogenetic lineages and their species coverage, protein lengths and protein similarities.
**Table S11** *MS5* homologs in *A. thaliana* and their subcellular localizations, expression profiles and adjacent transposon elements.
**Table S12** *Ka/Ks* ratio of homolog pairs between *A. thaliana* and *A. lyrata* in each homolog lineage.
**Table S13** Copy number determination of T_0_ transgenic Zhong6.
Please note: Wiley Blackwell are not responsible for the content or functionality of any Supporting Information supplied by the authors. Any queries (other than missing material) should be directed to the *New Phytologist* Central Office.
