# Supplementary material for: Evolution of the Brassicaceae‐specific MS5‐Like family and neofunctionalization of the novel MALE STERILITY 5 gene essential for male fertility in Brassica napus
Source: New Phytol. 2020 Nov 23;229(4):2339–56. doi: 10.1111/nph.17053 (PMC7894334; doi:10.1111/nph.17053)
Supplement: Supplementary file 4 — Fig. S1 Classical genetic model and three‐line hybrid breeding procedure of the genic male sterile system TE5ABC in B. napus. Fig. S2 Phylogenetic relationships of 23 Brassicaceae species belonging to four clades based on a published Brassicaceae phylogeny (Huang et al., 2016). Fig. S3 Maximum‐likelihood tree of MS5‐Like family inferred using 727 homologs which are divided into 25 homolog lineages (bootstrap values ≥60). Fig. S4 Maximum‐likelihood tree of MS5‐Like gene family inferred using 701 homologs (length ≥100 aa) which could be also divided into 25 homolog lineages (bootstrap values ≥60) as Fig. S3. Fig. S5 Synteny of the MS5 locus‐related genomic regions in nine Brassicaceae genomes and two outgroup species. Fig. S6 Nucleotide sequence alignment of 10 MS5 homologs/alleles and primers. Fig. S7 Gene frequencies of the MS5 locus in populations of B. napus and B. rapa. Fig. S8 Expression patterns and promoter activity of MS5 homologs. Fig. S9 Subcellular localization of BnMS5c and BrMS5a during early leptotene meiosis. Fig. S10 Western blot of bait or prey fusion proteins in yeast cells. [file NPH-229-2339-s004.pdf]

New Phytologist **Supporting Information**

Evolution of the Brassicaceae-specific *MS5-Like* family and neofunctionalization of the novel *MALE STERILITY 5* gene essential for male fertility in *B. napus*

Authors: Xinhua Zeng, Hao Li, Keqi Li, Rong Yuan, Shengbo Zhao, Jun Li, Junling Luo, Xiaofei Li, Hong Ma, Gang Wu, and Xiaohong Yan

Article accepted: 8 October 2020

The following Supporting Figures is available for this article:

Fig. S1 Classical genetic model and three-line hybrid breeding procedure of the genic male sterile system TE5ABC in *B. napus*.

Fig. S2 Phylogenetic relationships of 23 Brassicaceae species belonging to four clades based on a published Brassicaceae phylogeny (Huang *et al.*, 2016).

Fig. S3 Maximum likelihood tree of *MS5-Like* family inferred using 727 homologs which are divided into 25 homolog lineages (bootstrap values ≥60).

Fig. S4 Maximum likelihood tree of *MS5-Like* gene family inferred using 701 homologs (length ≥100 aa) which could be also divided into 25 homolog lineages (bootstrap values ≥60) as Fig. S3.

Fig. S5 Synteny of the *MS5* locus-related genomic regions in nine Brassicaceae genomes and two outgroup species.

Fig. S6 Nucleotide sequence alignment of ten *MS5* homologs/alleles and primers.

Fig. S7 Gene frequencies of the *MS5* locus in populations of *B. napus* and *B. rapa*.

Fig. S8 Expression patterns and promoter activity of *MS5* homologs.

Fig. S9 Subcellular localization of $BnMS5^c$ and $BrMS5^a$ during early leptotene meiosis.

Fig. S10 Western blot of bait or prey fusion proteins in yeast cells.

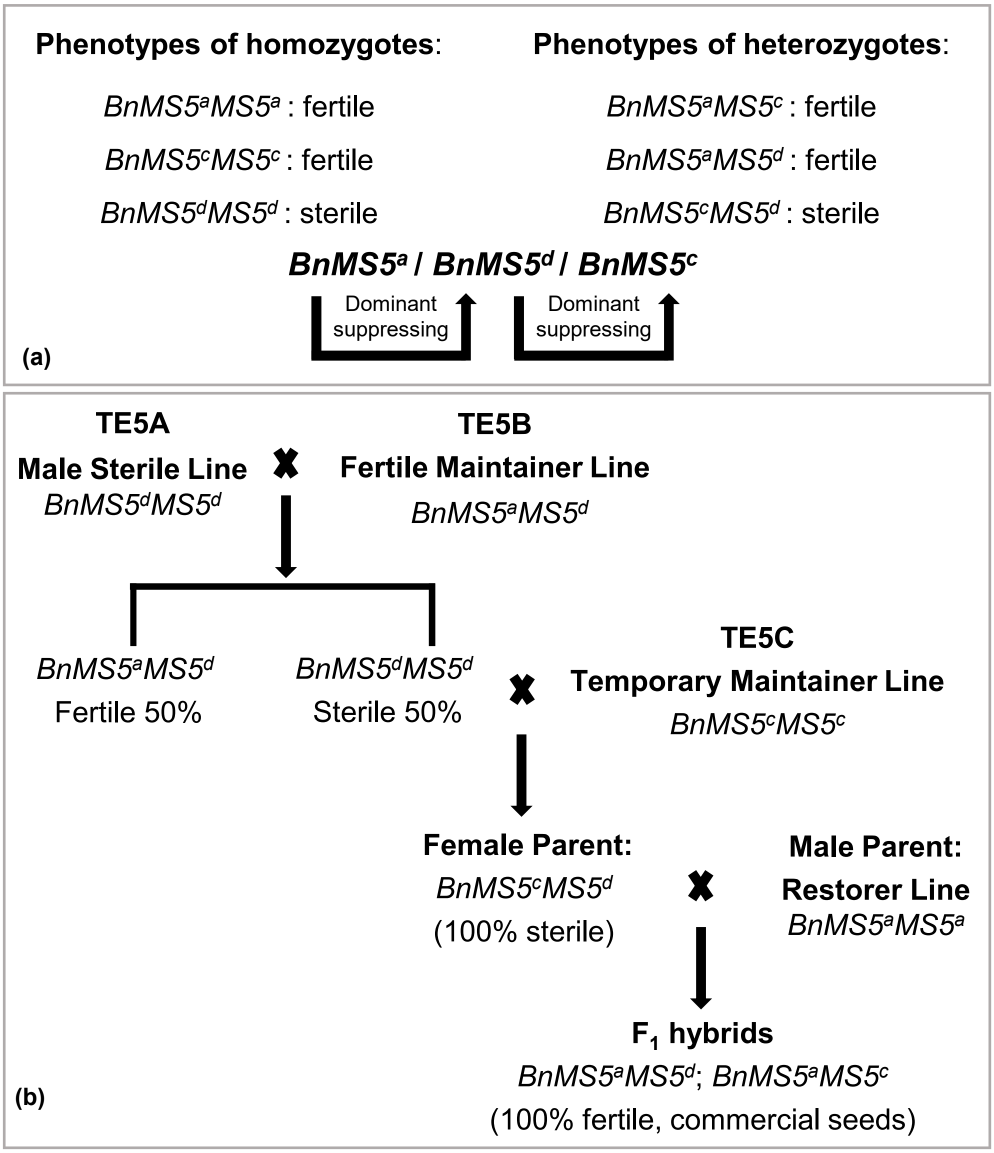

Fig. S1 Classical genetic model and three-line hybrid breeding procedure of the genic male sterile system TE5ABC in *B. napus*.

(a) The phenotypes of fertility and sterility of TE5ABC are controlled by multiple alleles: *BnMS5*^{a}, *BnMS5*^{c}, and *BnMS5*^{d}. *BnMS5*^{a} is the predominant suppressor gene relative to the male-sterile gene *BnMS5*^{d}, whereas *BnMS5*^{c} is a null allele gene. Curved blue arrows show the explicit-implicit relationships of these alleles: *BnMS5*^{a} > *BnMS5*^{d}> *BnMS5*^{c}.

(b) The genic male sterile breeding system TE5ABC is consisted of the male-sterile line TE5A (*BnMS5*^{d}*MS5*^{d}), fertile maintainer line TE5B (*BnMS5*^{a}*MS5*^{d}), temporary maintainer line TE5C (*BnMS5*^{c}*MS5*^{c}), and restorer line (*BnMS5*^{a}*MS5*^{a}). Firstly, male-sterile line TE5A is crossed with fertile maintainer line TE5B to generate a 1:1 ratio of fertile and sterile plants. Secondly, the sterile plants are crossed with temporary maintainer line TE5C to generate a 100% completely fertile population. This population could avoid the manual removal of 50% of the fertile plants in the field during hybrid seed production of a large area compared to when using other types of genic male sterile systems. Subsequently, the 100% sterile plants are extensively tested with a variety of restorer lines to produce superior hybrid seeds.

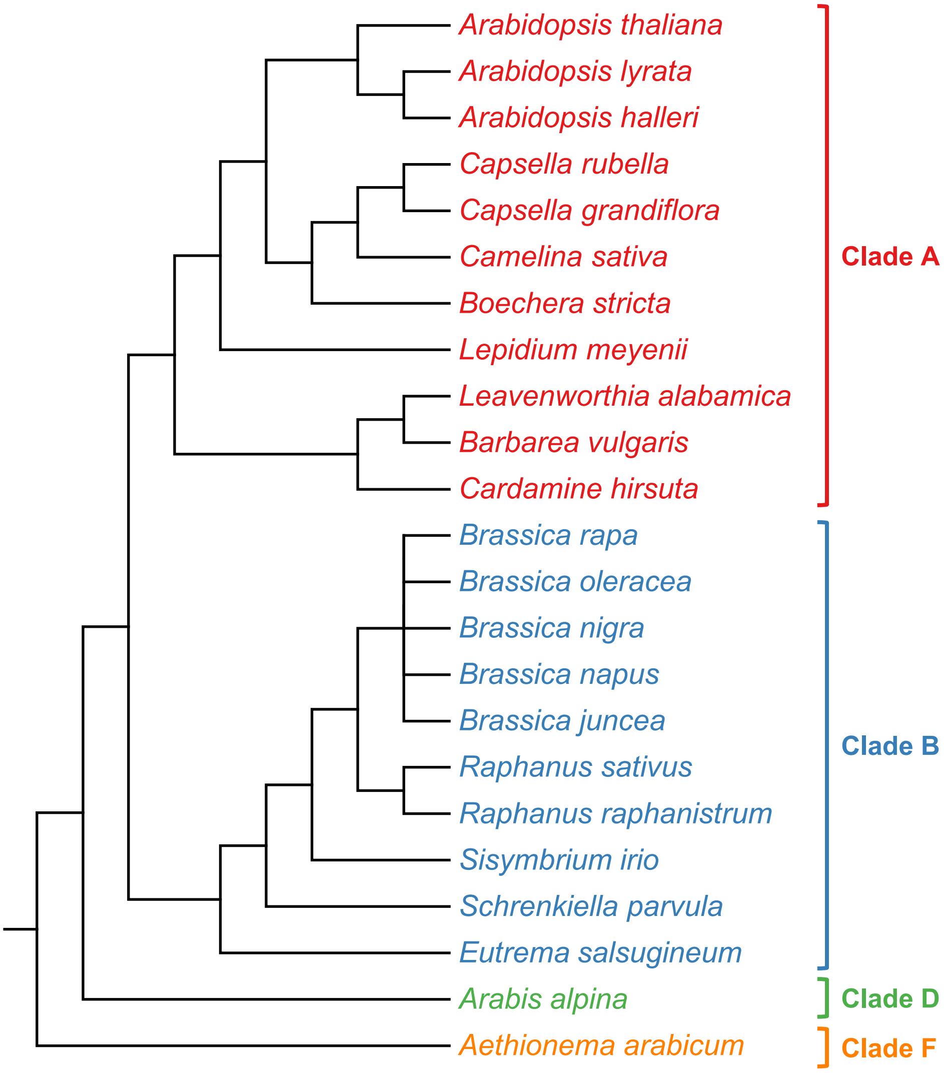

Fig. S2 Phylogenetic relationships of 23 Brassicaceae species belonging to four clades based on a published Brassicaceae phylogeny (Huang *et al.*, 2016).

Reference

Huang CH, Sun R, Hu Y, Zeng L, Zhang N, Cai L, Zhang Q, Koch M, Al-Shehbaz I, Edger PP *et al.* 2016. Resolution of Brassicaceae phylogeny using nuclear genes uncovers nested radiations and supports convergent morphological evolution. *Molecular Biology and Evolution* **33**: 394-412.

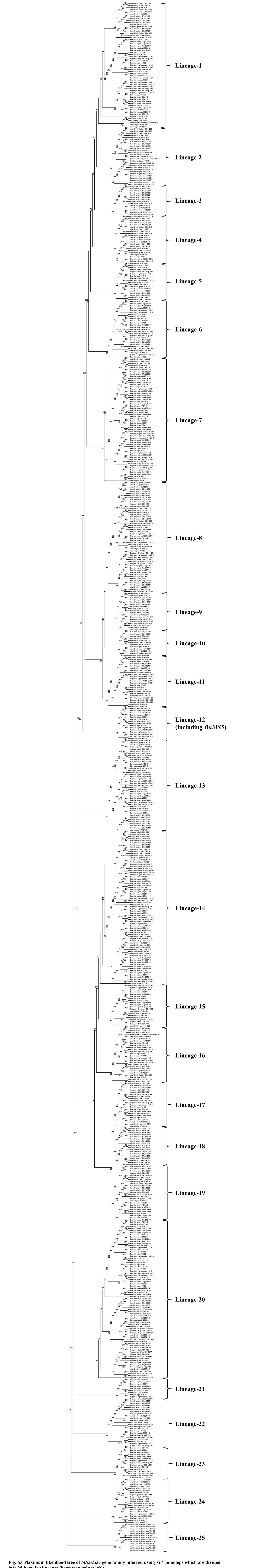

Fig. S3 Maximum likelihood tree of *MS5-Like* gene family inferred using 727 homologs which are divided into 25 homolog lineages (bootstrap values ≥60).
Bootstrap values (≥50) are shown for each node. Sequences are listed in Supporting Information Dataset S1, and alignment are shown in Supporting Information Dataset S2.

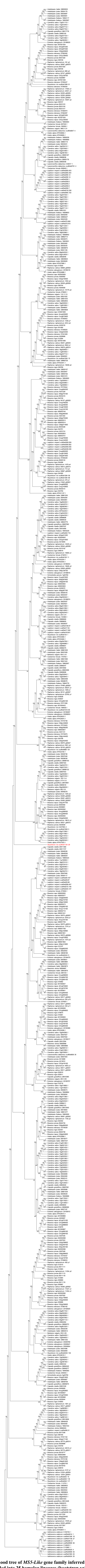

Fig. S4 Maximum likelihood tree of *MS5-Like* gene family inferred using 701 homologs (length ≥100 aa) which could be also divided into 25 homolog lineages (bootstrap values ≥60) as Fig. S3.
Bootstrap values (≥50) are shown for each node. Sequences are listed in Supporting Information Dataset S1.

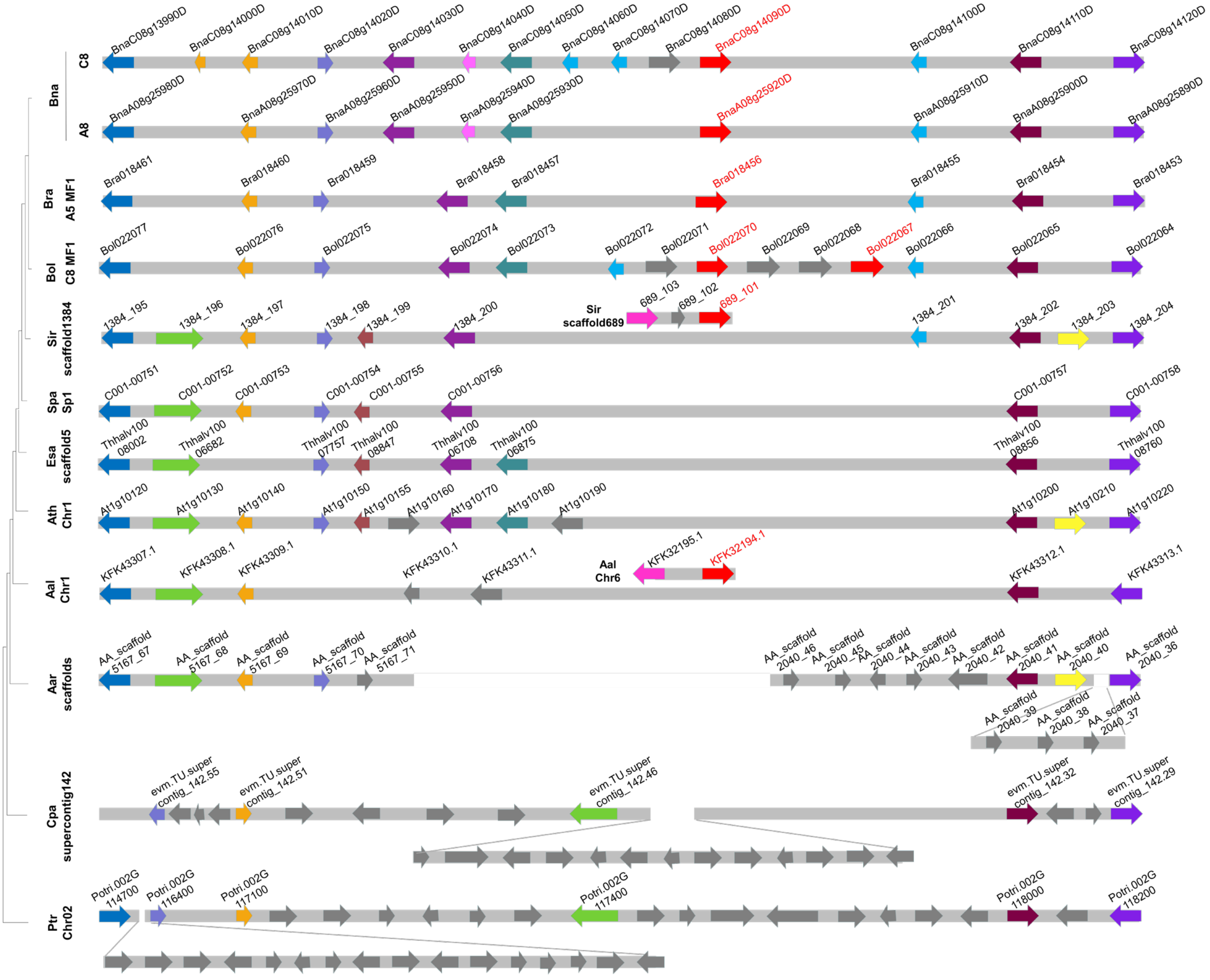

Fig. S5 Synteny of the *MS5* locus-related genomic regions in nine Brassicaceae genomes and two outgroup species.

Genes are indicated in arrows, in which red arrows are *MS5* homologs; and arrows in the same color represent syntenic homologs. Gray arrows indicate genes without any detected syntenic homologs in the corresponding regions of these genomes. Aal, *Arabis alpina*; Aar, *Aethionema araicum*; Ath, *Arabidopsis thaliana*; Bna, *Brassica napus*; Bol, *Brassica oleracea*; Bra, *Brassia rapa*; Cpa, *Carica papaya*; Esa, *Eutrema salsugineum*; Ptr, *Populus trichocarpa*; Sir, *Sisymbrium irio*; Spa, *Schrenkiella parvula*.

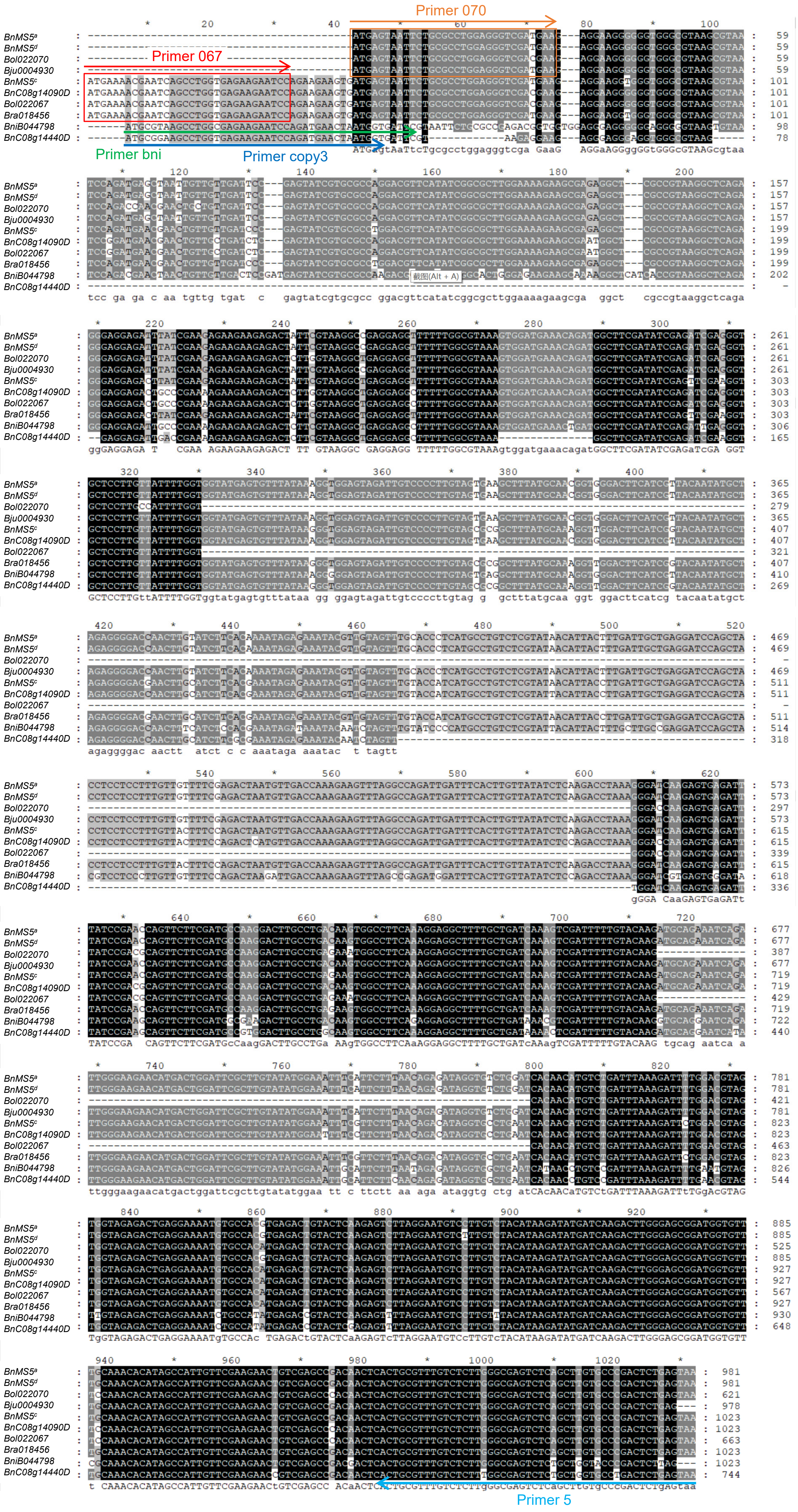

Fig. S6 Nucleotide sequence alignment of ten *MS5* homologs/alleles and primers.
Sequence alignment of the reported full *MS5* homologs from *Brassica* species. The primer pairs primer 067/primer 5, primer 070/primer 5, primer bni/primer 5 and primer copy3/primer 5 were used to isolate full *MS5* homologs in 22 diverse *Brassica* species accessions, respectively.

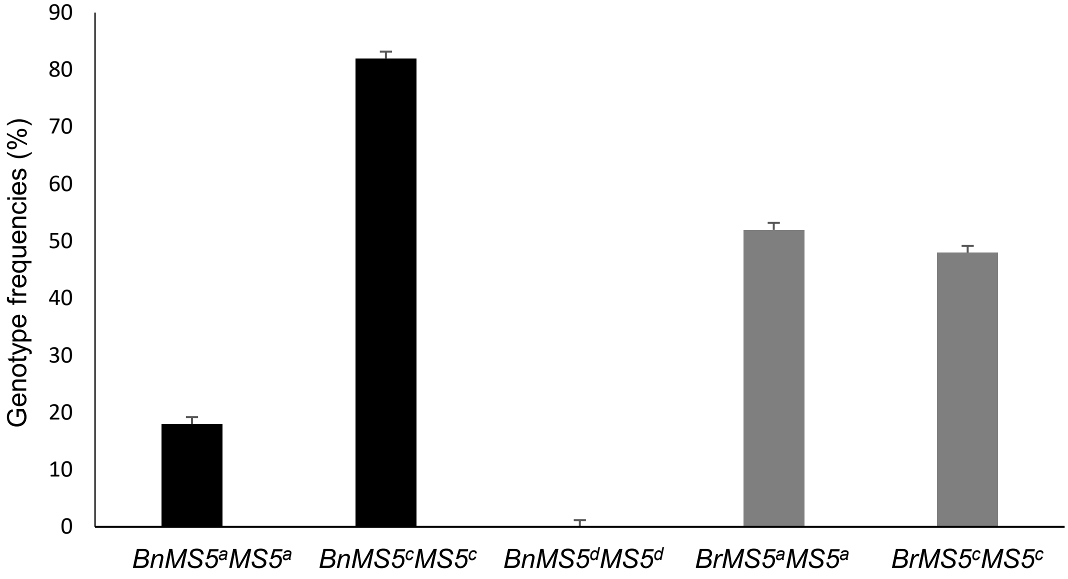

Fig. S7 Gene frequencies of the *MS5* locus in populations of *B. napus* and *B. rapa*. Data represent the mean ± SD.

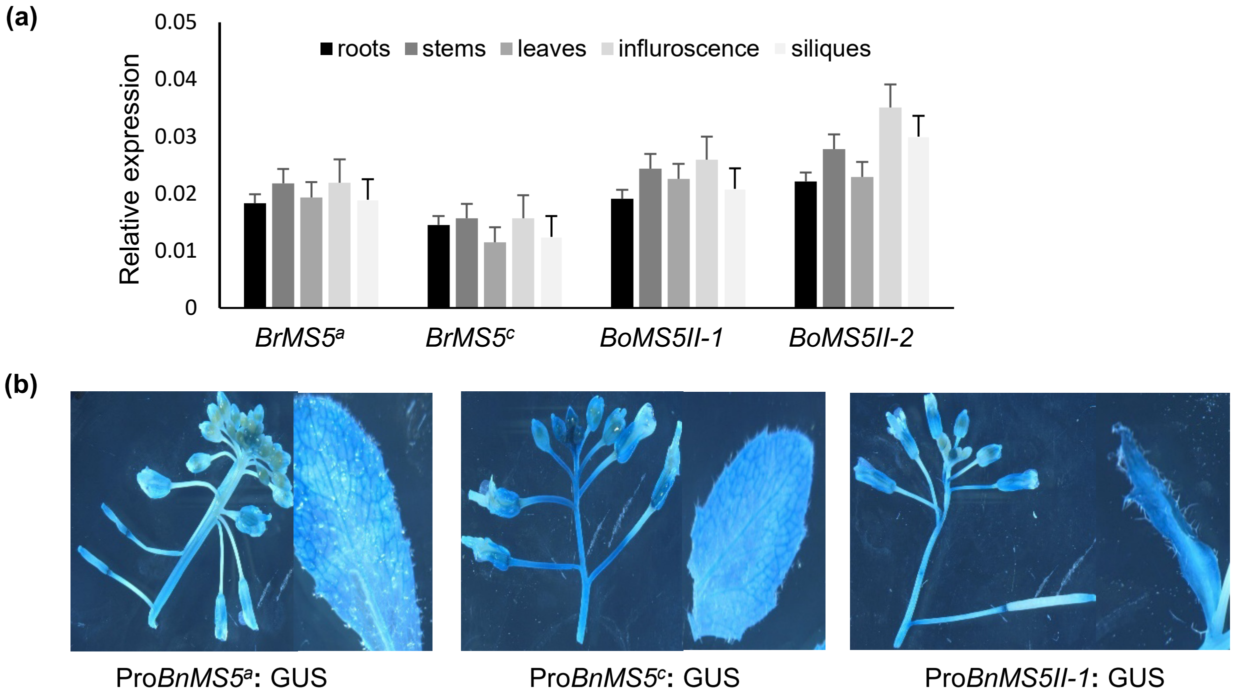

Fig. S8 Expression patterns and promoter activity of *MS5* homologs.

(a) Expression patterns of *MS5* homologs in *B. rapa* and *B. oleracea* using qPCR. The error bars indicate the standard deviation between three replicates of each plants with different *MS5* homologs.

(b) Histochemical analysis of the GUS activities (blue staining) directed by individual *BnMS5* promoters in transgenic *A. thaliana* plants. GUS expressions are driven by the three promoters *BnMS5*[a], *BnMS5*[c], and *BnMS5II-1*, respectively. At least three independent transgenic plants for each construct displayed similar GUS activities in different tissues, one of which is shown in each image.

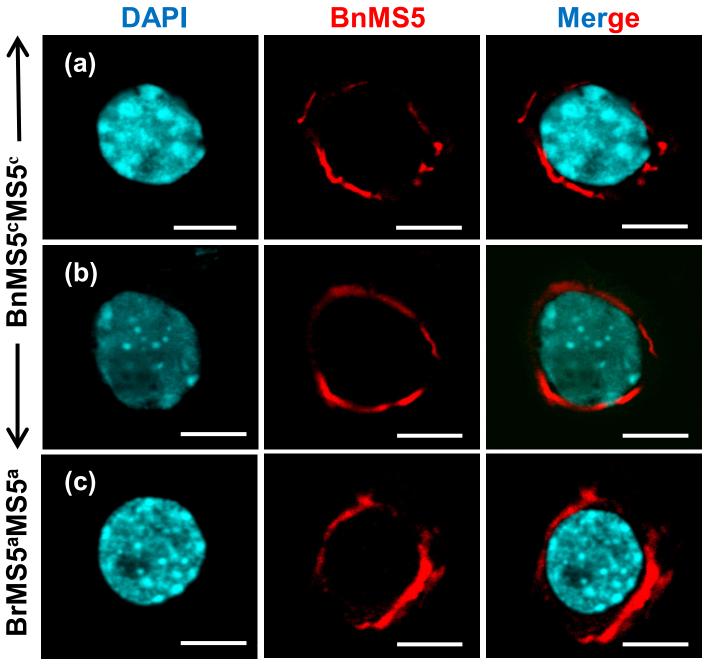

Fig. S9 Subcellular localization of BnMS5^{c} and BrMS5^{a} during early meiosis. Immunolocalization using rabbit polyclonal antibody against MS5 proteins (red) in plants carrying the *BnMS5^{c}* or *BrMS5^{a}* alleles. Chromosome DNA was counterstained with DAPI (white). Merged images show the overlap of red and white fluorescence. **(a) - (b)** *BnMS5^{c}MS5^{c}*; **(c)** *BrMS5^{a}MS5^{a}*. Bars = 10 µm.

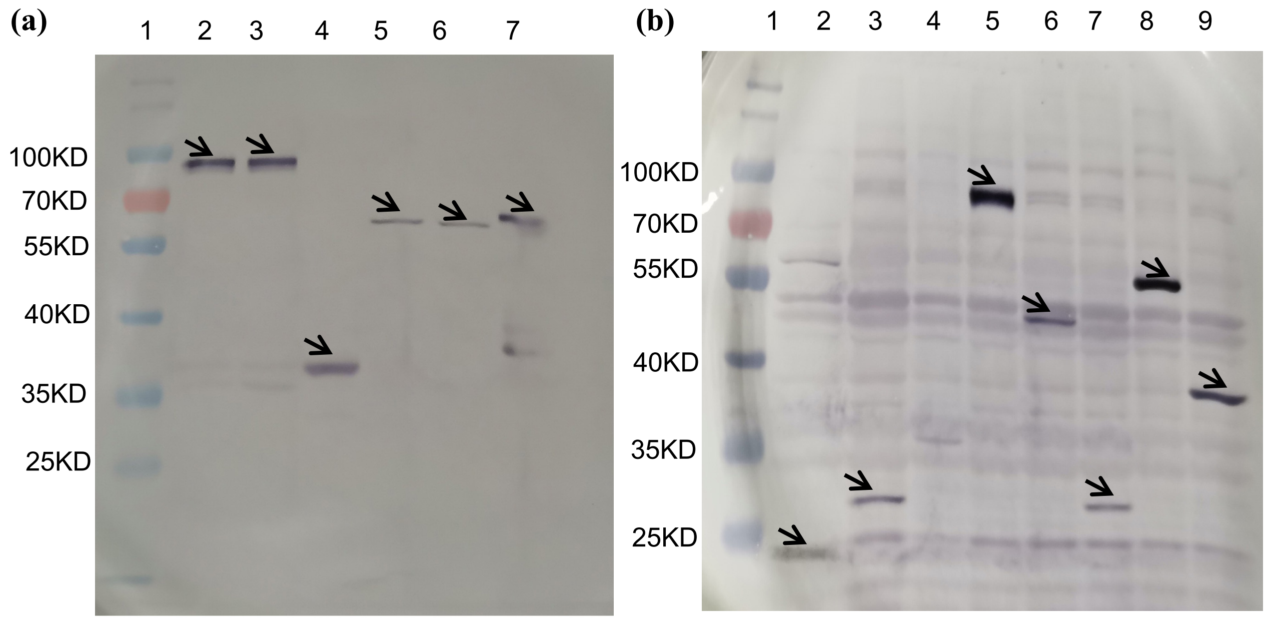

Fig. S10 Western blot of bait or prey fusion proteins in yeast cells.
BnMS5^{a}, BnMS5^{d}, BnMS5^{c}, and truncated BnMS5^{a} mutants B1, B2, B3, B4 and B5 were respectively constructed into the pGBKT7 plasmids as baits, encoding Gal4 BD fusion proteins. SUN1, SUN1Δ1, and SUN1Δ2 were respectively cloned into the pGADT7 vectors as preys, encoding Gal4 AD fusion proteins. Lam and p53 were constructed into the pGBKT7 plasmids, respectively, encoding the Gal4 BD fusion proteins. RecT was constructed into the pGADT7 plasmid, encoding the Gal4 AD-RecT fusion protein. The bait and prey constructs were transformed into Y2H Gold yeast cells and selected on the appropriate SD medium. Proteins were prepared using the Ura/SDS method. The blots were probed with GAL4 BD and GAL4 AD antibody (Clontech).
(a) Western blot of BnMS5^{a}, BnMS5^{d}, BnMS5^{c}, SUN1, SUN1Δ1, and SUN1Δ2 fusion proteins in yeast cells. Lane 1, protein marker with molecular weight indicated on the left; lane 2, SUN1Δ2-Gal4 AD (62KD); lane 3, SUN1-Gal4 AD (68KD); lane 4, SUN1Δ1-Gal4 AD (35KD); lane 5, BnMS5^{a}-Gal4 BD (58KD); lane 6, BnMS5^{d}-Gal4 BD (58KD); lane 7, BnMS5^{c}-Gal4 BD (60KD). Arrows indicate expected protein size.
(b) Western blot of B1, B2, B3, B4 and B5 fusion proteins in yeast cells. Lane 1, protein marker with molecular weight indicated on the left; lane 2, B1-Gal4 BD (24KD); lane 3, B3-Gal4 BD (33KD); lane 4, Negative control (Y2H Gold without containing vectors); lane 5, RecT-Gal4 AD (89KD); lane 6, B4-Gal4 BD (47KD); lane 7, B2-Gal4 BD (30KD); lane 8, p53-Gal4 BD (57KD); lane 9, Lam-Gal4 AD (40KD). Arrows indicate expected protein size.
